# Effect of the 35 nm and 70 nm Size Exclusion Chromatography (SEC) Column and Plasma Storage Time on Separated Extracellular Vesicles

**DOI:** 10.3390/cimb46050264

**Published:** 2024-05-06

**Authors:** Bernadett György, Krisztina Pálóczi, Mirjam Balbisi, Lilla Turiák, László Drahos, Tamás Visnovitz, Erika Koltai, Zsolt Radák

**Affiliations:** 1 Research Centre for Molecular Exercise Science, Hungarian University of Sport Science, Alkotás u. 42-48, 1123 Budapest, Hungary; gyorgy.bernadett.tf@gmail.com (B.G.); koltai.erika@tf.hu (E.K.); 2 Department of Genetics, Cell and Immunobiology, Semmelweis University, Üllői út 26, 1085 Budapest, Hungary; kikiczy@gmail.com (K.P.); visnovitz.tamas@med.semmelweis-univ.hu (T.V.); 3 Research Centre for Natural Sciences, Institute of Organic Chemistry, Magyar Tudósok Körútja 2, 1117 Budapest, Hungary; balbisi.mirjam@ttk.hu (M.B.); turiak.lilla@ttk.hu (L.T.); drahos.laszlo@ttk.hu (L.D.); 4 Department of Plant Physiology and Molecular Plant Biology, ELTE Eötvös Loránd University, Pázmány Péter sétány 1/c, 1117 Budapest, Hungary; 5 Faculty of Sport Sciences, Waseda University, Tokorozawa 2-579-15, Japan

**Keywords:** extracellular vesicles, size exclusion chromatography, human plasma, storage time, mass spectrometry

## Abstract

The technical difficulty of separating extracellular vesicles (EVs) from plasma proteins in human blood presents a significant hurdle in EV research, particularly during nano ultra-high-performance liquid chromatography–tandem mass spectrometric (UHPLC-MS/MS) analysis, where detecting “vesicular” proteins among abundant plasma proteins is challenging. Standardisation is a pressing issue in EV research, prompting collaborative global efforts to address it. While the MISEV guidelines offer valuable recommendations, unanswered questions remain, particularly regarding sample storage. We compared size exclusion chromatography (SEC) columns with pore sizes of 35 nm and 70 nm to identify fractions with minimal contaminating proteins and the highest concentration of small EVs (sEVs). Following column selection, we explored potential differences in the quality and quantity of sEVs isolated from platelet-free plasma (PFP) after long-term storage at −80 °C (>2.5 years) compared to freshly drawn blood. Our methodologically rigorous study indicates that prolonged storage, under correct storage and processing conditions, does not compromise sEV quality. Both columns effectively isolated vesicles, with the 70 nm column exhibiting a higher abundance of “vesicular” proteins. We propose a relatively rapid and moderately efficient protocol for obtaining a comparatively pure sEV fraction from plasma, facilitating sEV processing in clinical trials.

## 1. Introduction

Extracellular vesicles (EVs) are diverse membrane-enclosed particles in all cell types in the body. They contain a variety of biological molecules, including proteins, nucleic acids, and lipids, which can be transferred to other cells and tissues [1,2]. Based on their biogenesis, we distinguish two types of EVs: exosomes and ectosomes. Exosomes are of endosomal origin, while ectosomes are derived from the plasma membrane. The Endosomal Sorting Complex Required for Transport (ESCRT) proteins are intracellular proteins vital for the sorting and recycling of membrane proteins. They play a critical role in cellular processes such as cytokinesis and intracellular waste management. ESCRT proteins contribute to cytokinesis by engaging in membrane remodelling and abscission during cell division. Particularly, the components of the ESCRT-III complex aid in forming the cytokinetic bridge between daughter cells, which is crucial for their final separation. Additionally, ESCRT proteins facilitate the scission of the midbody, a transient structure formed during cytokinesis, by promoting membrane fission and vesicle release, thereby concluding the cell division process [3]. They also fulfill a crucial role in intracellular waste management by facilitating the degradation of cellular components through the endosomal–lysosomal pathway [4]. Their significance lies in maintaining cellular homeostasis and their association with a range of diseases. Typically, ESCRT proteins and their associated accessory proteins (such as Alix, TSG101, HSC70, and HSP90β) are commonly present in exosomes. This presence is attributed to the regulation of exosome formation and multivesicular body (MVB) transport by ESCRT proteins. Additionally, CD9, CD63, and CD81, which belong to the tetraspanin family, are proteins commonly found in EVs. These transmembrane proteins, along with other plasma membrane-associated proteins, are frequently identified in EVs [5,6,7]. EVs can be classified based on their size, with small EVs (sEVs) typically having a diameter of approximately 50–150 nanometers (nm). In addition to sEVs, cells also release medium-sized EVs (mEVs) ranging from 200 to 800 nm and large EVs with a diameter equal to or greater than 1000 nm [8].

EVs play crucial roles in both physiological and pathological processes, contributing to immune modulation, tissue repair, and the progression of cancer. Their diverse functions highlight their significance in cellular communication and disease mechanisms. In recent years, research on EVs has gained significant attention, and a growing body of evidence suggests that they hold great promise as a diagnostic and therapeutic tool [9].

There is no standard protocol for isolating EVs, but the most commonly used methods include differential centrifugation, density gradient centrifugation, and size exclusion chromatography (SEC) [10,11,12]. Polyethylene glycol (PEG) precipitation has also been used for isolating EVs from a variety of sources, including plasma, urine, and cell culture media [13,14]. However, PEG-based precipitation co-isolates non-EV contaminants, such as lipoproteins. While effective in isolating sEVs, PEG precipitation exhibits inferior purity compared to methodologies like SEC or sucrose density gradient centrifugation, thereby advising against its application for sEV isolation, particularly from protein-rich samples [15,16]. PEG molecules may suppress the ionisation of peptides of interest, resulting in decreased sensitivity and hindering the detection of low-abundance species. Additionally, PEG contamination can contribute to background noise in the mass spectrum, and PEG molecules may also form adducts with peptides, potentially complicating data interpretation [17]. Research on EVs is supported by the MISEV2018 and MISEV2023 guidelines, which provide directives for different aspects of EV separation and analysis [18,19]. The methodology employed in this article was devised and executed in accordance with the 2018 guideline, while the terminology adhered to the 2023 guideline. Furthermore, there are additional valuable resources for handling EVs, sourced from reputable references [20,21].

Proteomic analysis of EVs has become an essential tool for understanding the biological functions and mechanisms mediated by EVs. These proteomic studies provide valuable insights into the complex and diverse protein composition of EVs, their cargo, and their biogenesis. Numerous proteomic studies have explored the protein profiles of EVs released by different cell types under various physiological and pathological conditions [22,23,24,25,26,27]. This may involve investigating the impact of kidney transplantation on the composition of urinary extracellular vesicles [28] or exploring the potential utility of analysing donor-specific exosomes released during transplantation as a biomarker platform for rejection monitoring [29]. Additionally, conducting proteomic analysis of plasma exosome profiles in HIV patients and assessing their association with markers of immunological response and oxidative stress represents a significant advancement [30]. Furthermore, proteomic findings reveal promising candidate markers within extracellular vesicle populations derived from both pancreatic ductal adenocarcinoma (PDAC) and healthy control (HC) organoids, reflecting distinct tumorigenic or healthy states [31].

Human blood is frequently used in scientific studies due to its minimally invasive sampling procedure and the possibility to test many parameters from just a few millilitres of blood [32]. Proteins, nucleic acids, lipids, and other molecules present in blood can be analysed to identify indicators of specific diseases or physiological conditions. It is crucial to separate EVs from the blood samples in a way that guarantees sufficient purity and enables swift and efficient testing.

The aim of this study was to analyse sEV fractions that were separated from human PFP using 35 nm and 70 nm Izon qEV original SEC columns, as well as ultracentrifugation (UC). The SEC technique and the choice of qEV columns (Sepharose CL-2B resin) employed were grounded in findings from prior studies [11,33,34,35]. In our study, we wanted to work with a reproducible, reliable, and accessible method that could be used in the future, even in diagnostic fields. While several previous studies have compared different EV separation techniques, our focus was on separating sEVs for mass spectrometry (MS) to determine the most effective method for isolating a sufficient amount of low-contamination sEVs from human blood plasma. The ExoCarta database was used to define “vesicular” and “non–vesicular” proteins [36]. Additionally, we aimed to compare the quality of sEVs obtained from fresh PFP and PFP frozen for at least 2.5 years at −80 °C from the same people. Previous research has investigated the influence of extended storage on plasma or isolated EVs; however, it is pertinent to note that these investigations consistently encompassed shorter timeframes in comparison to the duration examined within our study [37,38].

## 2. Materials and Methods

### 2.1. Participants

Ethics permission was obtained from the Hungarian Ministry of Human Capacities (EMMI) in agreement with the Hungarian Scientific and Research Ethics Committee (ETT TUKEB, 25167-6/2019 EUIG). Eight physically active females (age; mean ± SD = 36.3 ± 8.9) and eleven physically active males (age; mean ± SD = 34.6 ± 10.4) participated in our study. Fasting blood sampling was used as a criterion, and healthy participants who performed weekly physical exercise (3–4 h) were selected. None of the volunteers had any known acute or chronic diseases.

### 2.2. Preparation of PFP from Whole Blood

**Figure 1:**
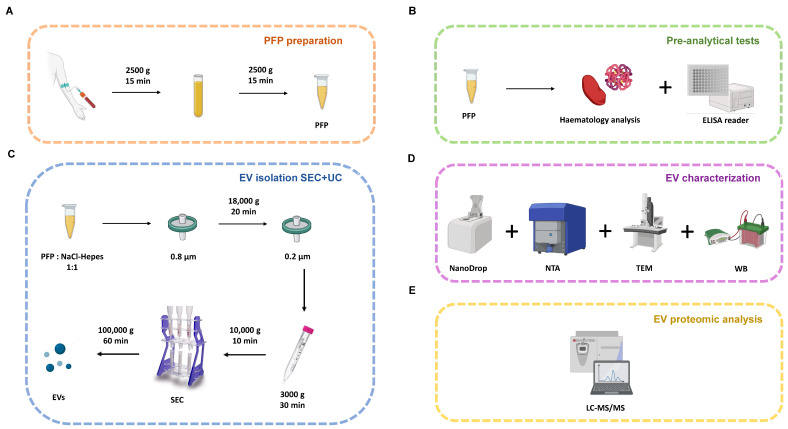
Schematic illustration of the workflow. (**A**) Preparation of PFP from whole blood. (**B**) The pre-analytical measurements of PFP. (**C**) EV isolation with SEC + UC. (**D**) EV characterisation methods. (**E**) EV proteomic analysis. EV: extracellular vesicles; PFP: platelet-free plasma; SEC: size exclusion chromatography; NTA: Nanoparticle Tracking Analysis; WB: Western blot; TEM: transmission electron microscopy; LC-MS/MS: liquid chromatography with tandem mass spectrometry.

The samples were collected by a qualified nurse at the Research Centre for Molecular Exercise Science (Hungarian University of Sport Science, Budapest, Hungary) over two different years. Frozen and fresh samples were obtained from the same person. Venous blood samples (16 mL) were collected from the cubital vein of subjects using two anticoagulant citrate dextrose-A (ACD-A) containing tubes via the BD Vacutainer blood collection system. Participants did not take medication at the time of sampling, and blood samples were taken after 12 h of fasting between 8 and 10 a.m. They were instructed to have a low-fat dinner as their final meal before their blood was drawn to avoid lipemic samples. The ACD-A blood collection tube was centrifuged at 2500× *g* for 15 min using an Eppendorf 5804R centrifuge (Eppendorf AG, Hamburg, Germany), and the resulting supernatant, which was the platelet-poor plasma (PPP), was aspirated into a 5 mL Eppendorf tube and centrifuged again at 2500× *g* for 15 min [39]. The resulting supernatant was PFP (Figure 1A), which, in the case of the 2020 samples, was snap-frozen in liquid nitrogen and stored at −80 °C until use. In the case of the blood samples taken in 2022, we processed the PFP immediately without freezing. In total, 12 samples were collected for analysis from frozen and fresh samples, and 21 samples were collected for comparison from 35 nm and 70 nm columns.

### 2.3. Pre-Analytical Measurements of PFP

Pre-analytical tests were conducted to assess the quality of the PFP and identify any factors that could interfere with the isolation process. Samples that appeared visually lipemic or haemolytic were excluded from further processing. Platelet and haemoglobin levels were measured using a Sysmex XE2100 haematology analyser (Symex Corp., Kobe, Japan), while absorbance was measured at various wavelengths using a LabSystems Multiskan ELISA (Thermo/LabSystems, Vantaa, Finland) reader (Figure 1B). This was crucial to avoid lipemic, haemolytic, or icteric samples that could interfere with EV isolation [40]. The following exclusion criteria were considered in the pre-analysis. Haemolysis was detected at 540 nm and, based on preliminary measurements, absorbances below 0.150 were deemed non-haemolytic. Using the haemolysis reference palette (https://www.cdc.gov/ncezid/dvbd/pdf/Hemolysis_Palette_Bookmark-P.pdf accessed on 1 June 2020), samples below 100 mg/dL were included. Icteric samples were measured at 450 nm and 492 nm wavelengths, with acceptance limits set at absorbances below 0.400 and 0.340, respectively. Lipemia was assessed at 620 nm and 690 nm wavelengths, and samples with absorbances below the limits of 0.150 and 0.120 were processed. Haemoglobin levels below 1 g/L were considered acceptable. If the sample exceeded a concentration of 5 million cells/L of platelets, it underwent an additional centrifugation step, as 5 million cells/L constituted the inclusion threshold. After filtration at 0.8 μm, it is recommended to remeasure the platelet count.

### 2.4. Isolation of sEVs

Each experimental procedure began with the isolation of sEVs from 2.5 mL of PFP. Collecting two tubes of blood per participant resulted in a surplus of PFP from each individual. Consequently, sEVs were isolated from a subset of participants for validation experiments, including WB, TEM, or MS.

In all cases, frozen samples were thawed at room temperature beforehand for about 1 h. The PFP samples (2.5 mL) were diluted to 5 mL with 0.2–0.1 µm tandem filtered NaCl-Hepes buffer and filtered through a 0.8 µm sterile Cellulose Acetate (CA) syringe filter via hydrostatic pressure to remove any remaining larger particles [41,42]. The filtered PFP samples were centrifuged at 18,000× *g* for 20 min (5810R Eppendorf centrifuge) to obtain the supernatant rich in sEVs. The supernatant was further cleaned using a CA syringe filter with a pore diameter of 0.2 µm and concentrated using a 100 kDa (Amicon Ultra-4 Centrifugal Filter Unit; Merck, New York, NY, USA) ultrafiltration tube (3000× *g* for 30 min; 5804R Eppendorf, Eppendorf AG, Hamburg, Germany) in order to concentrate samples to 1000–1500 µL. To ensure that samples had the same volume, they were filled up to 1500 µL with the buffer and centrifuged at 10,000× *g* for 10 min (Z216MK Hermle centrifuge, HermLe Labortechnik GmbH, Wehingen, Germany)) to remove any aggregates.

SEC can be used to remove proteins and lipoprotein particles from the plasma; however, this method only reduces the protein and “PLP” contamination of EV samples [34]. Two SEC columns with different pore sizes (35 nm and 70 nm qEV-original; IZON Science, Cambridge, MA, USA) were used in this study. For the comparison between frozen and fresh samples, a 70 nm pore size column was used, and the other samples were isolated in parallel using both 35 nm and 70 nm pore size columns. Samples (1500 µL) were applied to the SEC column, NaCl-HEPES buffer was used for elution, and fractions were collected as follows: waste fraction (3 mL), and then 1–10 fractions (0.5 mL/fraction). The pooled fraction containing sEVs was prepared from 1 to 3 fractions for the 35 nm column and 2–4 fractions for the 70 nm column. SEC fractions (1–8) and pooled samples were transferred to a UC centrifuge tube (#:344619; Beckman Coulter, Brea, CA, USA), filled with NaCl-Hepes buffer, and centrifuged at 100,000× *g* for 60 min (Type-100, Beckman Coulter centrifuge). After aspiration of the supernatant, the resulting pellet containing sEVs was resuspended in 15–20 µL of NaCl-Hepes buffer and stored at −80 °C until subsequent MS analysis (Figure 1C–E). The methodology outlined in our study for isolating sEVs was meticulously adhered to, and our step-by-step protocol is provided in Supplementary Table S1.

### 2.5. NTA

Particle size distribution and concentration were measured using a ZetaView Z-Nanoparticle Tracking Analysis instrument (Particle Metrix GmbH, Inning am Ammersee, Germany). It should be noted that this method cannot differentiate between “vesicular” and “non–vesicular” particles such as protein complexes, protein aggregates, and “PLPs” [43]. To determine the size distribution and concentration of sEVs from SEC fractions (1–8) and pooled fractions before UC, different dilutions (50–800×) were prepared. For pooled samples, those with a particle concentration below 1 * 10^9^ particles/mL were excluded from the analysis. The settings used for the samples included auto expose, gain: 28.8, offset: 0, shutter: 100, and sensitivity: 80. The measured data were analysed using the ZetaView Analysis software 8.05.10, and a minimum of 8 positions were used for analysis.

### 2.6. Spectrophotometry

The total protein content was measured using a NanoDrop ND-1000 instrument from Thermo Fisher Scientific (Waltham, MA, USA). The absorbance of the sample was measured at a wavelength of 280 nm, with NaCl-Hepes as the blank. We investigated fractions from 1 to 10. A sample volume of 1.5 µL was used, and the measurement was repeated three times. For samples extracted from the 35 nm and 70 nm columns, acceptance criteria included samples below a concentration of 0.75 mg/mL.

### 2.7. WB

After UC, the sEV pellet was resuspended in 30 µL of lysis buffer (CellLytic; Sigma, Darmstadt, Germany) containing protease inhibitors (cOmplete Protease Inhibitor Cocktail; Roche F. Hoffmann-La AG, Basel, Switzerland) while working on ice. To validate the protocol, SEC fractions (1–8) were individually analysed for both types of columns starting from 15 µL of protein lysate per sample well. Before gel electrophoresis, each sample was adjusted to 60 µL with the same lysis buffer, and 6 µL was used for micro-BCA measurement (Supplementary Table S2). Then, 16 µL of 5× Laemmli buffer (0.5 M Tris-HCL, 45 *v/v*% glycerol, 5 m/m% SDS, 0.25% bromophenol blue, 12.5% beta-mercaptoethanol) was added to the samples to denature the proteins at 95 °C for 5 min, and then the samples were applied to the gel (Criterion TGX 8–16%, 26 well; Bio-Rad, Hercules, CA, USA) wells. Electrophoresis was run in an ice-cooled running bath for 10 min at 170 V, followed by 60 min at 160 V. The proteins were then blotted (wet transfer, Trans-Blot^®^ Cell; Bio-Rad) onto polyvinylidene fluoride (Immun-Blot PVDF Membrane (0.2 µm pore size; Bio-Rad) membranes for 3.5 h at 80 V using methanol transfer buffer (25 mM Tris, 192 mM glycine, 20 *v*/*v*% methanol; pH 8.3). The proteins in the gels were stained with Sypro Ruby (Bio-Rad, Hercules, CA, USA) stain (fluorescent stains for total proteins) for 15 min on a rocker and washed twice for 5 min with 1× TBST (10× TBS 100 mL, MQ 900 m, TWEEN20 500 µL), after which photos of the gel were taken. The membranes were then blocked with SuperBlock (TBS) Blocking Buffer (Thermo Fisher Scientific, Waltham, MA, USA) solution at RT for 1 h. They were subsequently incubated overnight with rabbit and mouse anti-human primary antibodies at 4 °C, followed by goat polyclonal anti-rabbit and anti-mouse HRP-labelled secondary antibodies (Abcam, Cambridge, UK) for 30 min. The chemiluminescent signal was detected using Clarity MaxWestern ECL Substrate (Bio-Rad). Detection was performed using Imager ChemiDoc XRS+ Gel Imaging System (Bio-Rad, Hercules, CA, USA) and evaluation was performed using ImageLab (Bio-Rad software version 6.1). The antibodies used in this study are listed in Supplementary Table S3.

### 2.8. TEM

The visualisation of whole-mounted sEVs was conducted following the protocol outlined by Théry et al. [18]. We adapted established techniques with minor modifications for immunogold TEM [44,45]. EV solution (5 µL) was applied onto formvar-coated nickel grids (SPI Supplies, West Chester, PA, USA). Following a 10 min incubation at RT, excess liquid was eliminated by blotting with filter paper. EVs were immobilized by fixing them with 4% paraformaldehyde (PFA) for 10 min at RT. Subsequently, they were washed three times for 5 min each with distilled water. To prevent nonspecific binding, a solution of 2% sucrose (Molar Chemicals, Halásztelek, Hungary) in phosphate-buffered saline (PBS) was used for blocking, with an incubation period of 1 h at RT [44].

The primary antibodies were applied in 2% sucrose solution in PBS and incubated overnight at 4 °C. After three washes (5 min each) at RT with 2% sucrose solution, the secondary antibodies were applied in 2% sucrose solution for 1 h at RT. We opted for a 2% sucrose solution instead of bovine serum albumin (BSA) to prevent nonspecific protein binding to the formvar coat and minimize nonspecific immunoreactivity to BSA. Following the application of secondary antibodies, unbound antibodies were eliminated through three washes (5 min each at RT), and residual sucrose was removed by washing with PBS (three times for 5 min at RT). Subsequently, the samples were post-fixed using a 2% glutaraldehyde solution (Serva Electrophoresis GmbH, Heidelberg, Germany) and washed three times for 5 min at RT with distilled water [44]. Positive–negative contrasting was performed after immunogold labelling. The samples were analysed using a JEOL 1011 transmission electron microscope (JEOL, Tokyo, Japan). The diameters of EVs were measured utilising ImageJ software version 1.8.0 (*n* = 6 figures).

To detect the EV marker CD9 [18], a rabbit anti-CD9 IgG antibody (Abcam, Cambridge, UK) was applied. As a secondary antibody, polyclonal goat anti-rabbit IgG 5 nm gold (Sigma, Darmstadt, Germany) was employed. The antibodies used are listed in Supplementary Table S4.

### 2.9. MS

EV samples isolated from blood plasma were subjected to freeze–thaw cycles [46]. Next, 9× volume ice-cold ethanol was added to precipitate the proteins overnight at −20 °C. The pellet was washed twice and then re-dissolved in 20 µL 8 M urea in 50 mM ammonium bicarbonate. Dithiothreitol was added at 5 mM concentration and the solutions were incubated at 37 °C for 30 min, after which the samples were alkylated in the presence of 10 mM iodoacetamide at RT in the dark for 30 min. Solutions were diluted tenfold by 50 mM ammonium bicarbonate. In-solution digestion was performed first with Lys-C/trypsin mixture at a 1:100 weight ratio at 37 °C for 1 h, and then with trypsin at a 1:10 weight ratio at 37 °C overnight. Digestion was stopped by adding 1 µL formic acid, and samples were dried down and purified on a reversed-phase C18 spin cartridge, as described previously [47].

Samples were dissolved in 0.1% FA + 2% ACN solvent, and 1 µg of proteins was injected. The (A) series of measurements were performed on a Bruker Maxis II Q-TOF mass spectrometer with a CaptiveSpray nanoBooster ion source coupled to a Dionex Ultimate 3000 RSLC nanoUHPLC with an Acquity M-Class BEH130 C18 analytical capillary column [2]. MS operating parameters included a capillary voltage set at 1150 V, a gas pressure of 0.2 bar, and a drying gas flow rate of 3 L/min. Spectra were acquired in both MS and MS/MS modes across the mass range of 150–2200 *m*/*z*, with a cycle time of 2.5 s. MS spectra were obtained at a frequency of 3 Hz, while the acquisition rate for MS/MS spectra varied between 16 Hz and 4 Hz, depending on the precursor ion intensity. Collision energy was automatically adjusted by the control software based on the precursor ion’s *m*/*z* value and charge. Sodium formate served as the mass calibration standard, and recalibration of the data was performed using Compass DataAnalysis software version 4.3 (Bruker Daltonics, Bremen, Germany). For the (B) series of measurements, a Exploris 240 MS instrument and EASY-Spray ESI source were used with an EASY-Spray™ HPLC Column (Thermo Fisher Scientific, Waltham, MA, USA). ESI voltage was set to 1500 V, and the ion transfer tube temperature was at 250 °C. The data-dependent mode was used with a full MS precursor scan from 350 to 2000 *m*/*z* at a resolution of 60,000. Dependent MS/MS scans of the top 20 most intense precursor ions were carried out using 30% HCD collision energy, an isolation window of 1 *m*/*z*, and a resolution of 15,000. Before MS/MS scans, precursor ions were filtered based on intensity (1 × 10^3^) and charge state (2–4), and dynamic exclusion was also employed with a duration of 60 s. HPLC parameters for the (A) series of measurements were as follows: the sample trapping phase utilized a Thermo Fischer Scientific Acclaim PepMap100 C18 column (100 μm × 20 mm), followed by peptide separation on an Acquity M-Class BEH130 C18 analytical capillary column (1.7 μm, 75 μm × 250 mm, Waters, Milford, MA, USA). The flow rate was maintained at 0.3 μL/min, with solvents A and B consisting of 0.1% formic acid in water and in acetonitrile, respectively. In the gradient program, the proportion of solvent B was incrementally increased from 4% to 25% over 75 min, followed by a ramp to 40% over 15 min, and then to 90% over 1 min. The column was subsequently washed for 5 min and equilibrated with 4% B for 20 min. HPLC parameters for the (B) series of measurements were as follows: samples were injected into a pre-concentration column (Pepmap C18, 5 × 0.3 mm, 5 µm, Thermo Fisher Scientific, Waltham, MA, USA) with a 30 µL/min flow of 0.1% FA and 1% ACN for 3 min. Elution of the trapped peptides was carried out with 0.1% FA/H_2_O (A) and 0.1% FA/ACN (B) eluents at a flow rate of 250 nL/min onto the analytical column (PepMap RSLC C18, 50 cm × 75 µm, 2 µ). The initial composition of 3% B was linearly increased to 40% B in 120 min. The column was washed for 5 min with 90% B and re-equillibrated for 12 min using the initial 3% B composition. Peptides and proteins were searched against the Swiss-Prot human database using Byonic [48]. Mass tolerance was set as 10 ppm for precursor ions and 20 ppm for fragment ions, and 2 missed cleavage sites were allowed. Carbamidomethylation was considered fixed, while deamidation and oxidation were considered variable modifications. Proteins with a LogProb greater than 2 and at least 2 unique peptides were accepted. Quantification of proteins was performed using the LFQ algorithm in MaxQuant 1.6.17.0 software [49]. Potential contaminant proteins other than albumin were excluded from further analysis. Visualisation was carried out using Cytoscape 3.10.1. [50] PPI network was obtained by selecting experiments as the active interaction source with the highest confidence interaction score (0.9).

### 2.10. Statistical Analysis

Statistical analysis used to characterise the sEVs was performed with GraphPad Prism 9.4.1 software. We performed an Analysis of Variance (ANOVA) to compare variances across the means (or average) of different groups. Differences between 35 nm vs. 70 nm columns and fresh vs. frozen samples were evaluated using paired and unpaired Student’s *t*-test, and *p* < 0.05 was considered statistically significant.

### 2.11. EV-TRACK

We have submitted all relevant data of our experiments to the EV-TRACK knowledgebase (EV-TRACK ID: EV240007) (Van Deun J et al. EV-TRACK: transparent reporting and centralising knowledge in extracellular vesicle research. Nature methods. 2017;14(3):228–32). The submission of the experimental parameters to the EV-TRACK knowledgebase may be accessed and checked via the following URL: http://evtrack.org/review.php (accessed on 17 January 2024). Please use the EV-TRACK ID (EV240007) and the last name of the first author (György) to access our submission.

## 3. Results

### 3.1. Pre-Analytical Measurements of PFP

Of the 37 prepared PFP samples, 500 µL were set aside for pre-analytic measurements. Platelet counts (million cells/L; mean ± SD = 1.30 ± 2.09) and haemoglobin levels (g/L; mean ± SD = 0.41 ± 0.50) were initially measured using a haematology analyser (Sysmex XE2100). After performing 0.8 µm filtration during sEV isolation, the platelet count in PFP was measured using the same haematology analyser. The results showed that the platelet count was zero million cells/L for all samples (*n* = 37). As a second step, we used an ELISA plate reader that measured absorbances at five different wavelengths (450 nm, 492 nm, 540 nm, 620 nm, and 690 nm) to eliminate potential interferences from lipemia, haemolysis, and icterus during isolation. Haemolysis was detected at 540 nm (mean ± SD = 0.091 ± 0.033). Icteric samples were measured at 450 nm (mean ± SD = 0.343 ± 0.085) and 492 nm (mean ± SD = 0.257 ± 0.061) wavelengths. Lipemia was assessed at 620 nm (mean ± SD = 0.062 ± 0.023) and 690 nm (mean ± SD = 0.053 ± 0.018) wavelengths. Based on the results, we were able to isolate sEVs from each sample (*n* = 37).

### 3.2. Characterisation of sEVs Isolated Using SEC on a 35 nm or on a 70 nm Column

**Figure 2:**
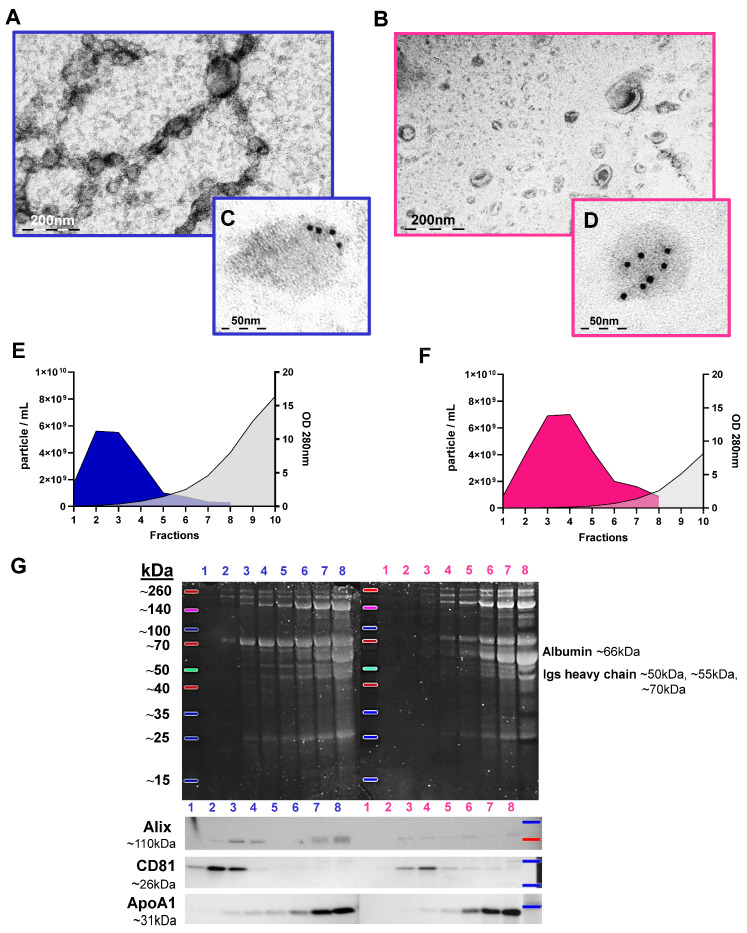
Characterisation of sEVs isolated using SEC on a 35 nm (blue) versus a 70 nm (pink) column. TEM images showing the morphology of sEVs after SEC pooled+UC: (**A**) 35 nm and (**B**) 70 nm. Immuno TEM images of sEVs after SEC pooled+UC: (**C**) 35 nm and (**D**) 70 nm. Average results of sEV characterisation isolated on a (**E**) 35 nm (*n* = 23) and a (**F**) 70 nm column (*n* = 29). The *X*-axis represents fractions, the left *Y*-axis shows NTA results in particle/mL, and the right *Y*-axis shows Nanodrop absorbance results at OD280. (**G**) Electrophoresis and Western blot results for total protein in sEV fractions (1–8) isolated with SEC + UC. SYPRO Ruby gel staining is shown in the upper image, with the left fractions belonging to the 35 nm column and the right fractions to the 70 nm column. Immunoblotting for the known EV markers (Alix and CD81) and the ApoA1 marker is displayed in the lower image.

We isolated sEVs from 31 out of 37 PFP samples using 35 nm and 70 nm columns. In total, 21 PFP samples were simultaneously isolated on both the 35 nm and 70 nm columns, with an additional 2 samples isolated exclusively in the 35 nm column and 8 samples isolated solely in the 70 nm column. We used transmission electron microscopy (TEM) to examine the morphology and size of sEVs (*n* = 6) in post-SEC ultracentrifuged and pooled samples for both the 35 nm (pool: 1–3; *n* = 210; min [nm] = 13.24; max [nm] = 542; mean [nm] ± SD = 98.594 ± 107.2) and 70 nm (pool: 2–4; *n* = 221; min [nm] = 17.58; max [nm] = 348.8; mean [nm] ± SD = 70.83 ± 47.96) columns, where min, max, and mean represent the diameter of the sEVs. Based on the particle counting of TEM and Nanoparticle Tracking Analysis (NTA) images, we distinguished three slightly overlapping populations: “plasma lipoprotein particles” (“PLPs”), sEVs, and mEVs (Supplementary Figure S1A–C). Supplementary Figure S1D presents the size distribution of frozen sEVs isolated on 35 nm and 70 nm columns, alongside freshly isolated sEVs on 70 nm columns, as assessed by NTA. PLPs are complex structures within the bloodstream tasked with transporting lipids, including cholesterol and triglycerides, throughout the body. We also performed morphological and immuno-TEM images with an anti-CD9 (Abcam, Cambridge, UK) EV marker antibody to label the EVs in the sample (*n* = 2) (Figure 2A–D). The NTA results were averaged and plotted (Figure 2E,F), revealing that for the 70 nm column (*n* = 29), the presence of plasma proteins was detectable from fraction 4 (absorbance > 0.100) and increased significantly, while for the 35 nm column (*n* = 23), plasma proteins were already present in fraction 2 (absorbance > 0.100). Combining the first 4 fractions, we found that the 70 nm column contained 1.24 times more particles/mL than the 35 nm column. In the last fraction (fraction 10), the average absorbance at 280 nm was 16.394 for the 35 nm column and 8.155 for the 70 nm column.

To further compare the two columns, we analysed the concentration of particles (particle/mL) and the absorbance at 280 nm for each fraction (Supplementary Figure S2C–F). We also utilised NTA to measure the Scatter Intensity, which we hypothesised to be linked to “PLP” contamination in the sample for both column fractions (1–6) (Supplementary Figure S2A,B).

Following SEC, UC was conducted on each fraction from 1 to 8, and after proper sample preparation (Section 2.7) and gel electrophoresis, the proteins in the gel were analysed by SYPRO Ruby gel or membrane staining (*n* = 8). Western blotting (WB) was performed for Alix and CD81 EV markers, as well as for the ApoA1 high-density lipoprotein (HDL) marker (Figure 2G). For the 35 nm column, the proteins in the samples were evident as early as the second fraction, whereas, for the 70 nm column, it was more likely to occur around the 3–4 fractions based on the gel staining. Supplementary Figure S2 shows the WB results for albumin (G), heavy chain immunoglobulins, and light chain immunoglobulin (H) in sEVs isolated from the 35 nm (1–4 fractions) and 70 nm (2–5 fractions) columns. The Alix EV marker was prominently visible in fractions 2–4 for the 35 nm column, whereas it appeared in fractions 3–6 for the 70 nm column. The slightly cloudy band seen in fractions 7 and 8 is thought to be an aspecific signal, as these fractions have high levels of plasma proteins such as albumin and other “PLPs”. The CD81 EV marker exhibited a strong signal in fractions 1–3 for the 35 nm column and in fractions 3–6 for the 70 nm column. To investigate the “PLP” content present in the sample, we examined the ApoA1, and a band appeared from fraction 3 for the 35 nm column and from fraction 4 for the 70 nm column.

**Figure 3:**
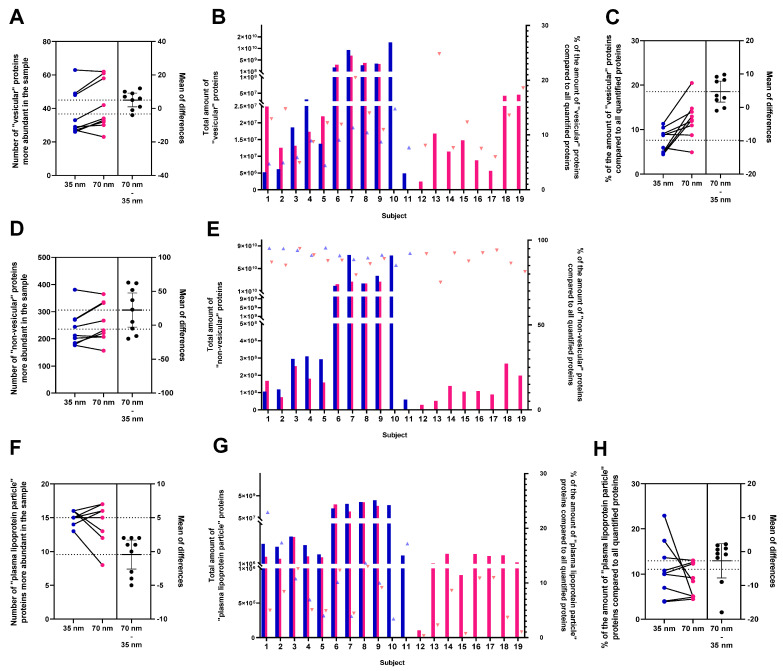
Analysis of MS data for sEVs isolated on 35 nm (blue) and 70 nm (pink) columns using SEC. Number of (**A**) “vesicular”(*p* = 0.0229; Paired *t*-test), (**D**) “non–vesicular”, and (**F**) “PLP” proteins from samples isolated with SEC pooled+UC. Lines connect samples from the same patients. The black dot indicates the effect size, the difference between the means of the paired data. The total amount of (**B**) “vesicular”, (**E**) “non–vesicular”, and (**G**) “PLP” proteins is shown in the left *Y*-axis (the data are indicated by columns), the right *Y*-axis shows the % of the amount of proteins compared to all quantified proteins (the data are indicated by triangles), and the *X*-axis represents the subjects. For the 9 pairs of samples, the Estimation Plot shows the results of the paired *t*-test of (**C**) “vesicular” (*p* = 0.0088), and (**H**) “PLP” compared to the total amount of protein.

**Figure 4:**
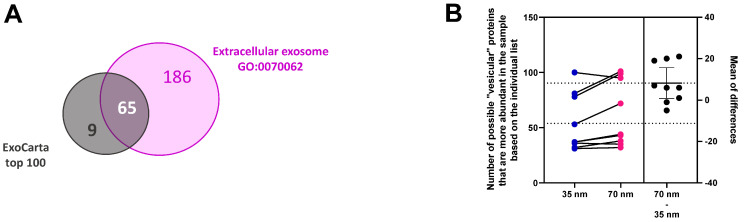
(**A**) The Venn diagram shows the comparison. (**B**) A paired *t*-test was executed on a list comprising 130 individual proteins for both the 35 nm and 70 nm columns (*p* = 0.0335). The sEVs isolated using SEC on a 35 nm column are marked in blue, and pink from the 70 nm column. Lines connect samples from the same patients. The black dot indicates the effect size, the difference between the means of the paired data.

MS-based proteomic experiments were performed on samples from nine subjects separated on both 35 nm and 70 nm SEC columns (*n* = 18). Additionally, for the purpose of quantification, 10 extra samples underwent analysis, comprising 2 samples from the 35 nm column and 8 samples from the 70 nm column. In the case of 35 nm and 70 nm SEC columns, pools of fractions 1–3 and 2–4 were used, respectively. Performing label-free quantitative proteomic analysis, a total of 74 proteins present among the top 100 exosome proteins and 539 “non–vesicular” proteins (not in the top 100 exosome proteins) were quantified. The results were compared pairwise (Figure 3A,C,D,F,H and Figure 4B) based on the number of proteins more abundant in one or the other sample, as well as on the basis of the total amount of quantified “vesicular”, “non–vesicular” and “PLP”. The number of quantified “vesicular” proteins ranged from 20 to 63, while the number of quantified “non–vesicular” proteins ranged from 114 to 381 for all samples investigated (Figure 3A,D). Six “vesicular” proteins (A2M, ANXA1, ANXA2, CD5L, GAPDH, and LGALS3BP) were quantified in all samples (Supplementary Table S5). The analysis revealed a higher amount of “vesicular” proteins compared to the total quantified proteins in the 70 nm column (Figure 3B), with percentages of 8.27% ± 3.18 in the 35 nm column and 12.31% ± 5.27 in the 70 nm column. The statistical analysis demonstrated a significant difference in all data with an unpaired *t*-test yielding a *p*-value of 0.0312 (Figure 3B on the right side). Furthermore, a paired *t*-test on nine pairs showed a trend towards significance with a *p*-value of 0.0088 (Figure 3C).

There were no differences in the number of “PLP” (APOA, APOA1, APOA2 APOA4, APOB, APOC1, APOC2, APOC3, APOC4, APOD, APOE, APOF, APOL1, APOM, and APOH) identified (Figure 3F). The analysis revealed a higher amount of “PLP” proteins compared to the total amount of proteins in the 35 nm column (Figure 3G), with percentages of 10.94% ± 6.34 in the 35 nm column and 6.68% ± 4.36 in the 70 nm column. The statistical analysis demonstrated a significant difference in all data with an unpaired *t*-test yielding a *p*-value of 0.0446 (Figure 3G on the right side). Furthermore, a paired *t*-test on nine pairs showed no significance (Figure 3H).

**Figure 5:**
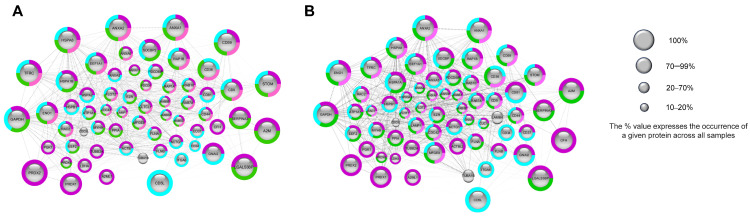
The cluster analysis of “vesicular” protein–protein interaction networks. We used the StringApp for Cytoscape software to retrieve protein networks from the STRING database. Protein–protein interaction networks were obtained by selecting “Experiments” with the highest confidence interaction scores (0.9). Each node represents a protein, and the dotted lines denote protein–protein interactions. The node sizes in the graph for “vesicular” proteins are proportional to the occurrence of protein identification across samples. The network is formed based on functionally enriched “GO Cellular Component” and “STRING Clusters,” with the main groups indicated by colours. Proteins of “vesicular” origin were isolated on (**A**) 35 nm and (**B**) 70 nm columns (the results exceeding 10% are presented).

Figure 5A,B show the “vesicular” proteins found for the 35 nm and 70 nm columns. Supplementary Figure S3 illustrates the ‘non–vesicular’ proteins, utilising the quantified proteins from the 35 nm column. STRING analysis of the “vesicular” proteins identified typical vesicle-related biological processes and cellular component GO annotation, such as “extracellular exosomes” (GO:0070062), “endomembrane system” (GO:0012505), “plasma membrane” (GO:0058865), and “bounding membrane of organelle” (GO: 0098588) (Supplementary Figure S4). Out of the 74 identified “vesicular” proteins, 71 were associated with an annotation. The plot displays only hits exceeding the 10% threshold (quantified in a minimum of two samples), revealing 62 proteins for the 35 nm column and 66 proteins for the 70 nm column (Figure 5A,B). The complete list of search results can be found in Supplementary Tables S5 and S7. On the other hand, network analysis performed on “non–vesicular” proteins showed that specific groups of proteins, e.g., “PLP” and immunoglobulins (“immunoglobulin complex” GO:0019814), were prominently present alongside “vesicular” proteins, indicating a biological relationship or analytical similarity between the groups. To enhance the clarity and simplicity of our figure, we only included immunoglobulin groups. However, all pertinent proteomic data are readily accessible for download in the data availability statement section. Among all proteins, “non–vesicular” proteins (Figure 3E) constituted 91.73% ± 3.04 of proteins for the 35 nm column and 87.69% ± 5.12 for the 70 nm column (out of which 48.72% ± 19.08 and 44.21% ± 16.15 were immunoglobulins in the case of the 35 nm and 70 nm columns, respectively) (Supplementary Figure S2I). The second most abundant group of proteins, categorized as “plasma lipoprotein particles” (GO:0034358), accounted for 11.82% ± 6.35 and 7.51% ± 4.62 of the “non–vesicular” proteins in the case of the 35 nm and the 70 nm columns, respectively. Additionally, proteins associated with the “complement cascade” (CL:18846) and “haemostasis, and dissolution of Fibrin Clot” (CL:18731) were also identified. In addition, in the figure, we have highlighted the components in pink that may be part of the EV corona, e.g., the lipid bilayer, which is the defining component of EVs (“bounding membrane of organelle” GO:0098588) and albumin (highlighted in black). Albumin connects to the EV surface, becoming an integral component of the cargo. However, it concurrently presents as a significant contamination of plasma origin in substantial amounts. The specific proportion between the two is not known. Although the ExoCarta top 100 includes albumin, we have included it separately in the analysis for the reasons mentioned above. The plasma membrane proteins and cell surface proteins are denoted in light blue. Of note, the EV corona components have not yet been listed among the ‘vesicle’ proteins of the ExoCarta top 100.

We compared the proteins identified by ExoCarta top 100 with those associated with “extracellular exosomes” (GO:0070062), as outlined in Supplementary Table S5. The comparative analysis is depicted in a Venn diagram (Figure 4A). A total of 186 proteins were obtained that were included in the GO list (Supplementary Table S6). The initial list underwent further refinement by excluding proteins associated with “PLP”, immunoglobulin, haemostasis, and the complement cascade system. Subsequently, this narrowed list was subjected to testing across the nine pairs of samples. Additionally, proteins with a single occurrence in both columns were excluded, resulting in a final set of 130 proteins, constituting our individual list (Supplementary Table S7). A paired *t*-test conducted on these 130 proteins (Figure 4B) revealed a higher hit rate in the 70 nm column (*p* = 0.0335).

### 3.3. Characterisation of sEVs Separated by an SEC 70 nm Column Both for Fresh and Frozen PFP

We set to compare sEVs separated from frozen PFP (*n* = 6) stored at −80 °C for >2.5 years with those isolated from freshly drawn blood (*n* = 6). For the frozen sample data (*n* = 6), the 70 nm results of the comparison of the two types of columns were used. Based on our previous comparison of the 35 nm and 70 nm columns, which showed more efficient sEV isolation with less lipoprotein contamination in the 70 nm column, we isolated EVs collecting fractions 1–8 using the 70 nm column for this comparison [51].

**Figure 6:**
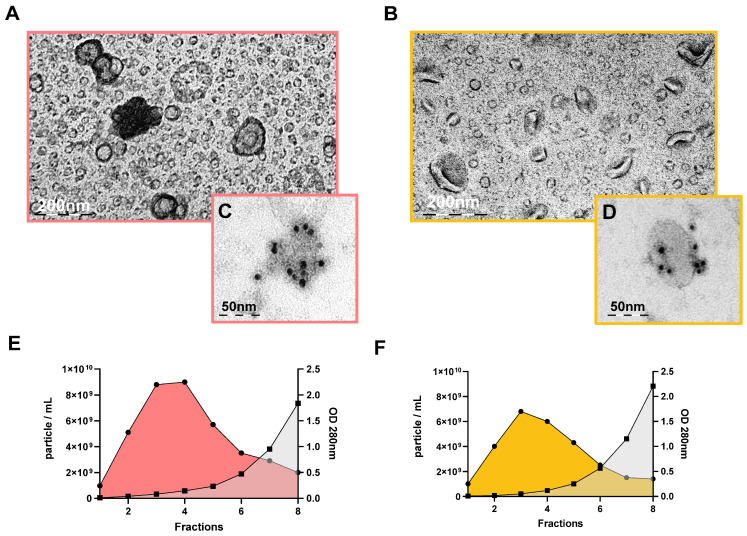
Characterisation of sEVs isolated using SEC 70 nm column in both frozen (orange) and fresh (yellow) PFP samples. TEM images displaying the morphology of sEVs from (**A**) frozen and (**B**) fresh samples after SEC pooled+UC. Immuno TEM images of sEVs from (**C**) frozen and (**D**) fresh samples after SEC pooled+UC. Average results of sEV characterisation isolated from (**E**) frozen (*n* = 6) and (**F**) fresh (*n* = 6) samples with SEC. The *X*-axis represents fractions, the left *Y*-axis shows NTA results in particle/mL, and the right *Y*-axis shows Nanodrop absorbance results at OD280.

TEM was used to examine the sEVs from frozen (pool of 2–4 fractions) and fresh (pool of 2–4 fractions) samples, and the results are presented in Figure 6A–D. In this case, UC was also used after SEC. The particle number in the samples was quantified using NTA (particle/mL), while the protein absorbance was measured at 280 nm using a NanoDrop instrument. The data are presented in Figure 6E,F.

We compared the frozen (*n* = 5) and fresh (*n* = 6) samples by measuring the number of particles in each fraction using NTA. We used Nanodrop to measure the absorbance of the proteins at 280 nm both in frozen (*n* = 6) and fresh (*n* = 6) samples (Supplementary Figure S5C–F). No difference was observed between the two sample types. Similarly, we found no difference in the Scatter Intensity measured in fractions 1–6 between frozen (*n* = 5) and fresh (*n* = 5) samples (Supplementary Figure S5A,B).

**Figure 7:**
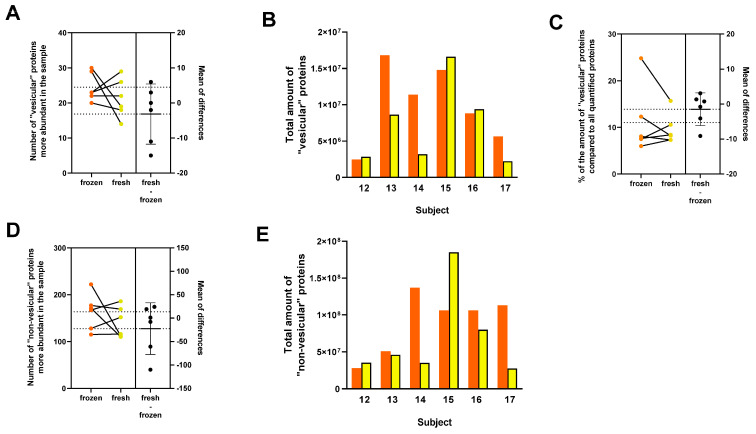
Analysis of MS data for sEVs isolated using SEC 70 nm column in both frozen (orange) and fresh (yellow) PFP samples. Lines connect samples from the same patients. The black dot indicates the effect size, the difference between the means of the paired data. (**A**) Number of “vesicular” and (**D**) “non–vesicular” proteins from frozen and fresh samples isolated with SEC+UC. Lines connect samples from the same patients. (**B**) Comparison of the total amount of “vesicular” and (**E**) “non–vesicular” proteins from frozen and fresh samples isolated with SEC pooled+UC. (**C**) For the 6 pairs of samples, the Estimation Plot shows the results of the paired *t*–test of “vesicular” proteins compared to the total amount of proteins (*p* = 0.4595).

Fresh and frozen samples from six subjects were investigated and compared. A total of 35 “vesicular” and 279 “non–vesicular” proteins were quantified. The number of “vesicular proteins” varied between 14 and 30, while the number of “non–vesicular” proteins varied between 110 and 222 per sample (Figure 7A,D).

Figure 7B,E show the total amount of “vesicular” and “non–vesicular” proteins in six pairs of samples. A paired *t*–test on six pairs showed no difference in “vesicular” proteins compared to the total amount of proteins (Figure 7C). We noted a difference in one pair of data; nevertheless, the overall comparison indicates no distinction between fresh and frozen samples. The results suggest that there is no clear relationship between the proteomic profile and the origin of the sample (fresh vs. frozen). Therefore, frozen samples are equally suitable for further analysis.

## 4. Discussion

In this study, a total of 37 human blood plasma samples were collected from volunteers. Significant attention was paid to the participants’ health status, and there was a 12 h fasting period prior to sample collection, synchronized timing of sample collection, plasma preparation, and pre-analytical measurements [52].

In terms of methodology, we followed the protocol established by the International Society on Thrombosis and Haemostasis (ISTH) for the preparation of PFP [39,53,54]. At the beginning of the EV separation process, we implemented a 0.8 µm filtration step [32,44], followed by a platelet count determination using a haematology analyser. The results indicated that no detectable level of platelets remained in the samples, with a platelet count of 0 × 10^6^ cells/L for all samples (*n* = 37).

Overall, obtaining PFP is important in isolating EVs from human blood to ensure the purity and specificity of the EV population being studied and to avoid confounding effects from platelets. The assessment of haemolysis and lipemic conditions in plasma is crucial during the isolation of EVs from human blood. Haemolysis, the breakdown of red blood cells, can contaminate EV samples and affect downstream analyses. Lipemic plasma, characterized by high lipid levels, can interfere with EV isolation and characterisation. By evaluating and addressing these conditions, researchers can ensure the integrity and reliability of EV preparations for accurate analysis [54]. We considered it essential to incorporate a pre-analytical step prior to isolation, a practice emphasized in the MISEV2023 guideline [19].

In our study, we initially compared the quantity and quality of sEVs isolated on 35 nm and 70 nm columns using TEM, NanoDrop, NTA, and WB methods, and the identification of “vesicular” and “non–vesicular” proteins using MS analysis. Böing et al. [35] demonstrated the purification of vesicles from human PFP through Sepharose CL-2B SEC, a method supported by our own investigation. We selected Izon qEV original columns for our study which are filled with Sepharose CL-2B resin. Our choice was guided by the aim of obtaining the purest sEV fraction with minimal “PLP” contamination from plasma. Additionally, we prioritized the use of a standardized, commercially available column with a well-established track record to establish our methodology. This was also facilitated by preliminary literature research. Sidhom et al. [11] conducted a review confirming SEC as a swift, reproducible, and relatively pure technique for fractionating EVs. Furthermore, Baranyai et al. [33] noted that vesicles isolated from blood plasma via SEC exhibited a significant albumin-free fraction, albeit with a reduced vesicle yield. Stranska et al. [55] demonstrated that SEC provides EVs relatively promptly and with notably elevated purity.

In our results of comparing 35 nm and 70 nm columns, we observed that the presence of cup-shaped sEVs in TEM images (Figure 2A,B and Figure 6A,B) confirmed the presence of sEVs in the samples. The sample preparation was carried out by Théry et al. [45]. The discrepancy observed between cup-shaped and relatively circular vesicles in the TEM photos is attributed to technical variations in sample preparation, resulting in a contrast variation between samples. We also immunolabelled sEVs with a CD9 vesicle marker-specific antibody (Figure 2C,D and Figure 6C,D) to identify them. The results obtained from NanoDrop and NTA analyses (Supplementary Figure S2C–F) revealed significant differences in the absorbance measured at OD 280 nm between the two columns for all 10 fractions. The fractions collected from the 70 nm column consistently exhibited lower protein content. Ter-Ovanesyan et al. [56] investigated three methodologies (SEC, UC, and ExoQuick) for isolating EVs from plasma and cerebrospinal fluid (CSF) samples. Unlike our study, they observed that the 35 nm column provided the purest fraction for EVs, contrasting with the 70 nm column. However, it is noteworthy that their analysis solely focused on the albumin-to-EV ratio, whereas we conducted proteomic analysis and Albumin, ApoA1 WB, on the samples. In the NTA results (particle/mL), significant differences between the two columns were observed in five out of eight fractions. The sEVs appeared to elute more gradually from the 70 nm column, necessitating a slightly different pooling scheme, where fractions 1–3 were pooled for the 35 nm column, while fractions 2–4 were pooled for the 70 nm column. The WB results supported our previous observations. Notably, visual differences were observed in SYPRO Ruby gel staining after gel electrophoresis between the two columns (Figure 2G). Proteins began to appear in the 2nd fraction for the 35 nm column, while this occurred around the 3rd-4th fractions for the 70 nm column. A similar trend was observed for the appearance of the ApoA1 marker in the fractions. The pooling of samples (35 nm = 1–3 fractions, 70 nm = 2–4 fractions) was determined based on the WB results. Fernández-Rhodes et al. [57] utilized SEC-coupled ultrafiltration for the isolation of EVs from cell culture. Their WB findings closely resembled ours, showing a stronger presence of the ApoA1 lipoprotein marker in the later fractions, while the CD9 and Annexin A2 vesicle markers were more prominent in the earlier fractions. Ekström et al. [58] observed vesicle marker CD63 and Flotillin-1 enrichment in early fractions and ApoA1 predominance in later fractions of vesicles isolated using SEC from lymphatic drainage fluid. In conclusion of this section, the presence of Alix and CD81 EV markers in the fractions also verified our pooling method. It is crucial to acknowledge that, owing to biological variance, even fraction 5 from the 70 nm column may contain vesicles (Supplementary Figure S2F).

For the MS results, we also used the Venn diagram (Figure 4A) to compare proteins from ExoCarta top 100 and “extracellular exosomes” (GO:0070062), illustrating shared (65 proteins) and distinct proteins. In the comparison of the nine pairs (Figure 3A), for both the ExoCarta top100 (*p* = 0.0229) and our individual list (Figure 4B), the count of “vesicular” proteins was higher in the 70 nm column (*p* = 0.0335). The analysis unveiled a greater amount of “vesicular” proteins (Figure 3B) in comparison to the overall quantified proteins in the 70 nm column (*p* = 0.0312).

It is essential to note that some proteins co-isolate with EVs due to their adherence to the EV surfaces, such as certain immunoglobulins [2], apolipoproteins [59], and albumin, which may play a protective role [60]. A protein corona is formed around EVs in blood plasma [2]; in this study, these corona proteins are classified as proteins of “non–vesicular” origin.

Based on our own results and other studies [61,62], we selected the 70 nm column to compare frozen (>2.5 years) and fresh (same day) sEVs isolated from PFP samples from the same individuals as an important part of the investigation.

In the second part of the investigation, we did not find any differences in the TEM images between frozen and fresh PFP samples (Figure 6A–D). When comparing the 35 nm and 70 nm columns, we found that the examination of up to eight fractions was sufficient, as fractions above eight could not be measured by NTA (the instrument detected no traces), and therefore, we did not collect them further. Neither NTA nor NanoDrop results showed any differences (Supplementary Figures S1 and S5C–F) between sEVs isolated from frozen and fresh PFP samples, which was further supported by the MS data. Similarly to our findings, Yuana et al. [37] observed no differences in the size of frozen and fresh EVs as determined by Nanoparticle Tracking Analysis (NTA). Kabagwira et al. [38] deduced that the extended freezing of EVs does not induce alterations in the EV size. Our MS data suggest no correlation between the proteomic profile and the origin of the sample (fresh vs. frozen).

Yuana et al. [37] investigated the effect of freezing on PFP for 1 year and used centrifugation for EV isolation. Kabagwira [38] and co-workers froze unprocessed plasma for 16 months and used the ExoQuick method for EV isolation. In contrast, we froze pre-analytically detected PFP for at least 2.5 years and found no significant differences between frozen and fresh samples. Therefore, we conclude that PFP samples stored for a prolonged period (>2.5 years) are equally suitable for sEV isolation as freshly prepared PFP samples. These results may help in designing experiments for studies where, for some reason, it is not possible to process samples immediately or to take all samples at the same time or in close proximity to each other.

Summarising the entirety of the study, despite WB suggesting more noticeable differences in the purity of preparations, the MS data did not align with those observations. Additionally, there was no statistical significance observed in the MS data for “PLPs”, albumin, and immunoglobulins. For the nine pairs of samples, it was verified that the number of “vesicular” proteins was more abundant in the 70 nm column. We conclude that both the 35 nm and 70 nm columns are suitable for vesicle isolation; however, our recommendation leans towards the use of the 70 nm column, as indicated by our findings. It is not usually possible to process clinical samples immediately, so it is important to know whether stored samples can be trusted. We found that adequately prepared PFP can be safely stored at −80 °C for a period of up to 2.5 years.

Our working group is currently developing a protocol for the separation of mEVs and sEVs from the same sample (manuscript in preparation by Panna Királyhidi).

It should also be noted that at the time of our study’s inception, the newer version of the Izon qEV gen2 column has not been available yet. Considering Izon Science’s continuous development of SEC columns, future studies could give researchers new insights into the isolation of sEV by these newly released columns and similar methods.

Data discussed in this article may contribute to the search for improved protocols to separate EVs from “PLPs” and other proteins as efficiently as possible. Another intention of the authors was to help reduce the artefacts in EV-related studies and the associated misinterpretation and increase the reliability of EV research.

## 5. Conclusions

In this study, we isolated sEVs from the blood plasma of healthy subjects using our developed protocol. We assessed the efficacy of isolation using IZON qEV original 35 nm and 70 nm columns, with detailed proteomic analysis. The results showed that both columns effectively isolated vesicles, with the 70 nm column demonstrating a higher abundance of “vesicular” proteins. Comparing vesicles isolated from the same subject’s fresh plasma and frozen plasma stored for >2.5 years in the 70 nm column revealed no significant differences, indicating the suitability of frozen plasma samples for vesicle analysis. The limiting factors of this method include the need for a relatively large amount of sample (minimum 8 mL of blood for one type of test) and the need for adequate equipment, e.g., ultracentrifuge. Another limitation is that the isolate obtained is not very specific and is not 100% pure. Our results offer a foundation for further detailed investigations and provide a robust basis for the methodology needed for functional experiments on vesicles.

## Data Availability

Proteomics data are available in the MassIVE repository under the https://doi.org/doi:10.25345/C59K4642X link and can be downloaded via FTP (ftp://massive.ucsd.edu/MSV000093783/ accessed on 1 Februry 2024).

## Supplementary Materials

The following supporting information can be downloaded at: https://www.mdpi.com/article/10.3390/cimb46050264/s1, Figure S1: Characterization of pooled sEVs isolated using SEC; Figure S2: Characterization of sEVs isolated using SEC on a 35 nm versus a 70 nm column; Figure S3: The cluster analysis of “non–vesicular” proteinprotein interaction networks; Figure S4: The Ancestor Chart displays the Cellular Component term used for the String; Figure S5: Characterization of sEVs isolated using SEC 70 nm column in both case of fresh and frozen PFP; Table S1: Step by step protocol; Table S2: MicroBCA results for Western blot; Table S3: Antibodies for Western blot, which used in the study; Table S4: Antibodies for TEM, which used in the study; Table S5: Table containing the "vesicular" proteins identified through quantification using the ExoCarta top 100 list across all samples; Table S6: Proteins not included in the ExoCarta top 100 list are annotated under “Extracellular exosome GO:0070062”; Table S7: The protein list is derived from potential EV proteins identified in the 9 pairs.

## Abbreviations

ESCRT—Endosomal Sorting Complex Required for Transport; Evs—extracellular vesicles; HDL—high-density lipoprotein; ISTH—International Society on Thrombosis and Haemostasis; LC-MS/MS—Liquid Chromatography with tandem mass spectrometry; MVB—multivesicular body; NTA—Nanoparticle Tracking Analysis; UC—ultracentrifugation; UHPLC-MS/MS—ultra-high-performance liquid chromatography–tandem mass spectrometric; PEG—polyethylene glycol; PFP—platelet-free plasma; PLP—plasma lipoprotein particles; SEC—size exclusion chromatography; TEM—transmission electron microscopy; WB—Western blotting.
