# Supplementary material for: Effect of the 35 nm and 70 nm Size Exclusion Chromatography (SEC) Column and Plasma Storage Time on Separated Extracellular Vesicles
Source: Curr Issues Mol Biol. 2024 May 6;46(5):4337–57. doi: 10.3390/cimb46050264 (PMC11120626; doi:10.3390/cimb46050264)
Supplement: Supplementary file 1 [file cimb-46-00264-s001.zip › cimb-2955937-supplementary-Figures.pdf]

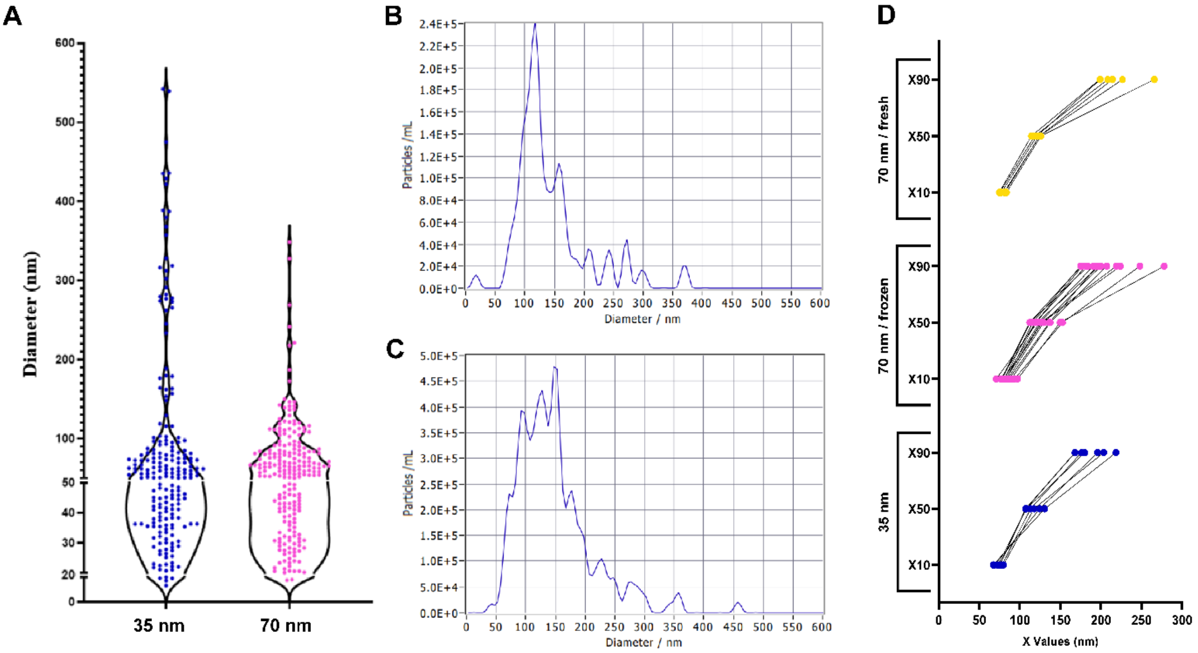

Supplementary Figure S1. Characterization of pooled sEVs isolated using SEC on a 35 nm (fraction 1–3) versus 70 nm (fraction 2–4) columns. (**A**): The size distribution of sEVs and particles isolated on 35 nm and 70 nm columns. The diameter of sEVs was measured from TEM images using ImageJ software. X–axis shows the populations for the 35 nm and 70 nm columns, y–axis shows the size distribution (nm). (**B**): NTA image of the pooled fraction of sEVs and particles isolated from the 35 nm and (**C**): the 70 nm column. (**D**): The NTA size distribution (X10, X50, X90) of frozen sEVs isolated on 35 nm (n=6) and 70 nm (n=13) columns, alongside freshly isolated sEVs on 70 nm (n=6) columns.

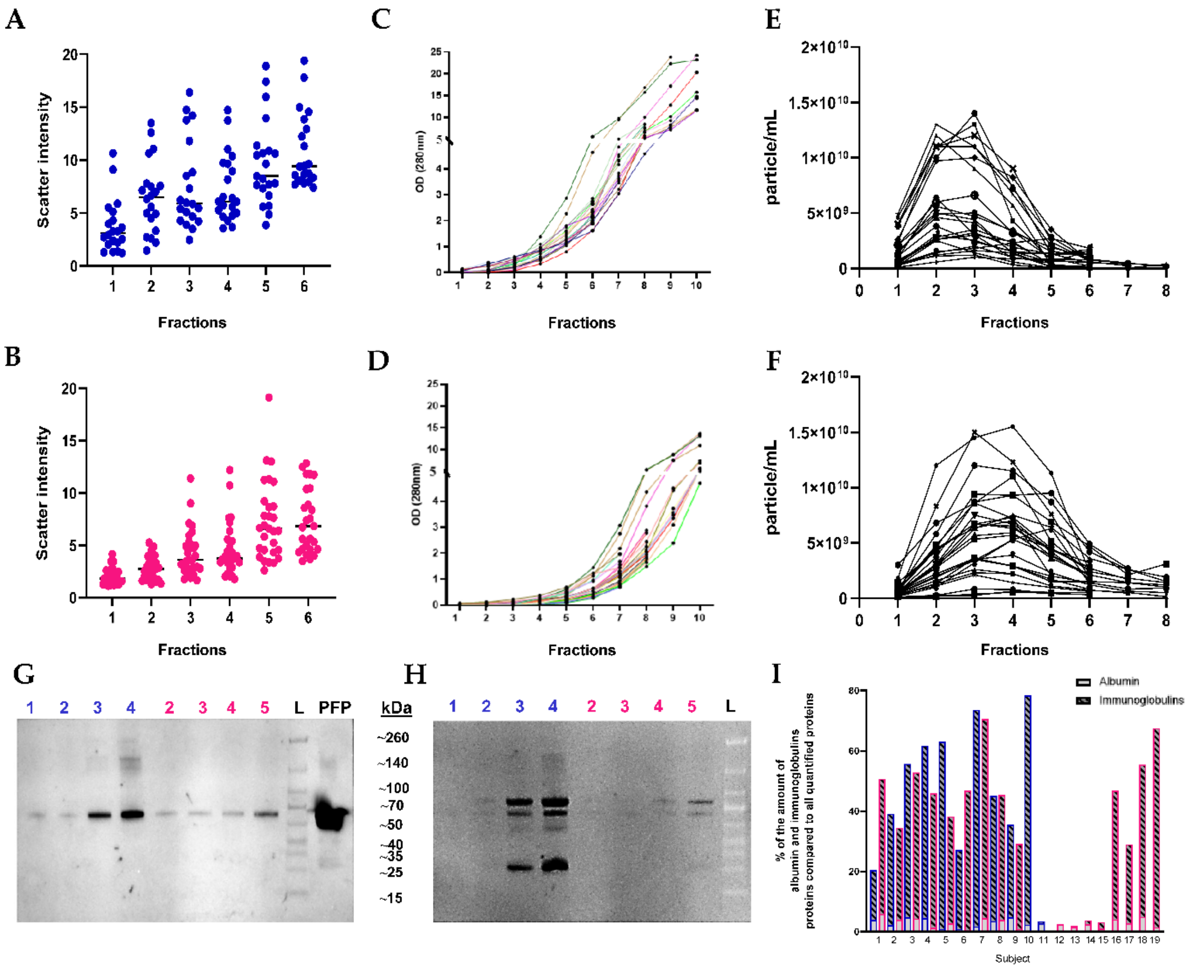

Supplementary Figure S2. Characterization of sEVs isolated using SEC on a 35 nm **(A,C,E)** versus a 70 nm **(B,D,F)** column. (**A–B**): The x–axis of the graph displays fractions 1–6, while the y–axis represents the Scatter Intensity (SI), a parameter measured by the NTA (ZetaView) instrument. We found a significant difference for all 6 fractions (**p < 0.01: fraction 3,5; ***p < 0.001: fraction 1,2,4; ****p < 0.0001: fraction 6). (**C–D**): The x–axis of the graph displays fractions 1–10, while the y–axis represents the absorbance measured on 280nm with Nanodrop. We found significant differences in the absorbance at 280nm for all 10 fractions (*p < 0.05: fraction 1; ***p < 0.001: fraction 2–9; ****p < 0.0001: fraction 10). (**E–F**): The concentrations of sEV fractions (particle/mL) measured with the NTA instrument are plotted on the y–axis and the fractions 1–8 on the x–axis. We found significant differences in the concentration of particles in 5 out of 8 fractions (*p < 0.05: fraction 1,2,7; **p < 0.01: fraction 5,6). (**G**): The WB results for albumin, (**H**) heavy chain immunoglobulins and light chain immunoglobulin in sEVs isolated from the 35 nm (1–4 fractions) and 70 nm (2–5 fractions) columns. (**I**): The x–axis represents subjects, the y–axis shows the % of the amount of albumin and immunoglobulin proteins compared to all quantified proteins. Blank part of the columns represent albumin, and striped part of the columns indicate immunoglobulins. Samples isolated from the 35 nm column are indicated in blue and from the 70 nm column are indicated in pink.

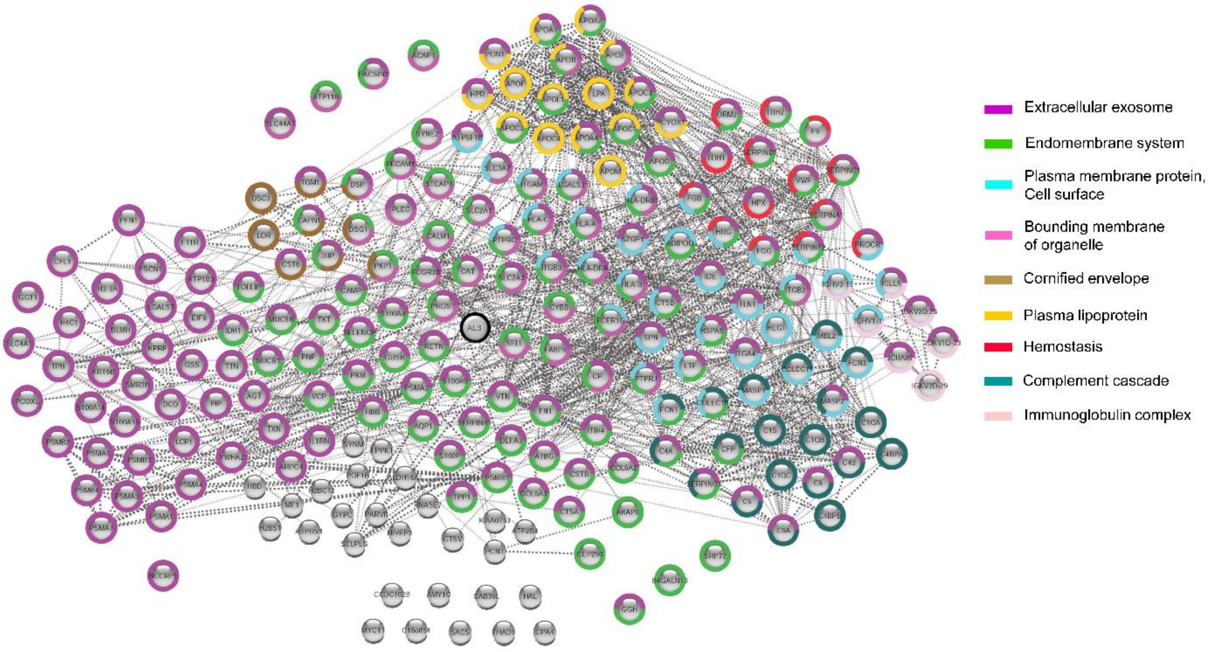

Supplementary Figure S3. The cluster analysis of "non−vesicular" protein−protein interaction networks. We used the StringApp for Cytoscape softwares to retrieve protein networks from the STRING database. Protein−protein interaction networks were obtained by selecting “Experiments” with the highest confidence interaction scores (0.9). Each node represents a protein, and the dotted lines denote protein−protein interactions. The network is formed based on functionally enriched "GO Cellular Component" and "STRING Clusters," with main groups indicated by colours. The figure shows the proteins of ”non−vesicular” origin isolated on 35 nm column (results exceeding 20% are presented).

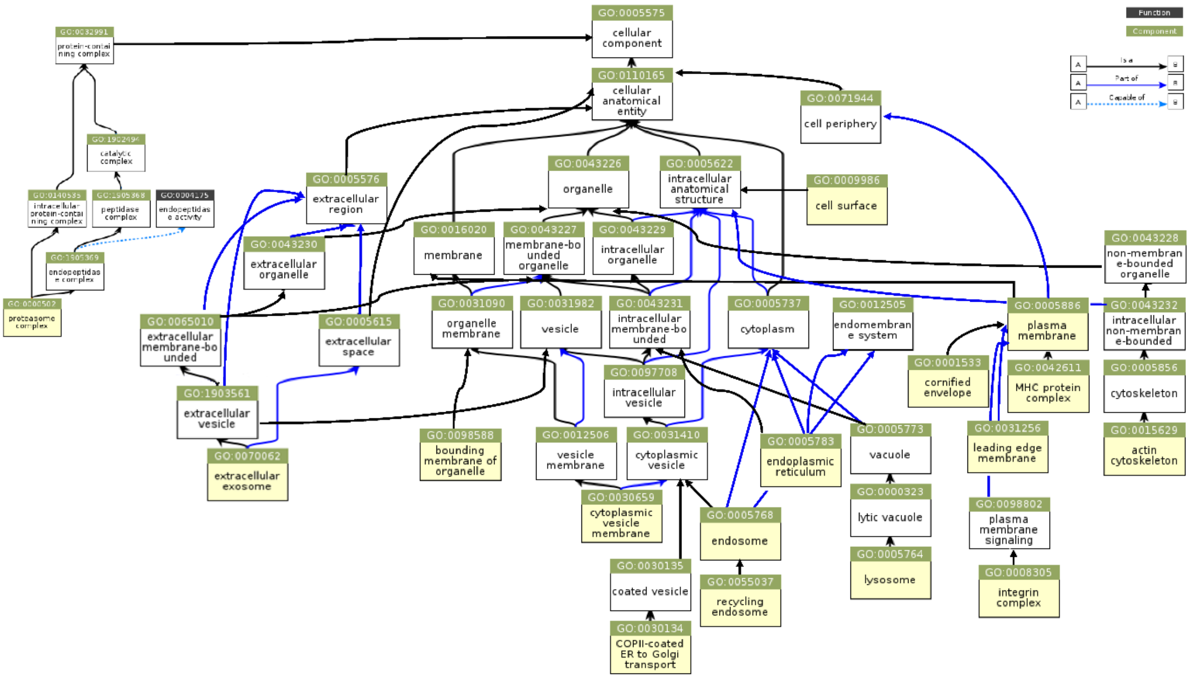

Supplementary Figure S4. The Ancestor Chart displays the Cellular Component term used for the String, sometimes including associated child terms in certain cases. Source information from: https://www.ebi.ac.uk/QuickGO/.

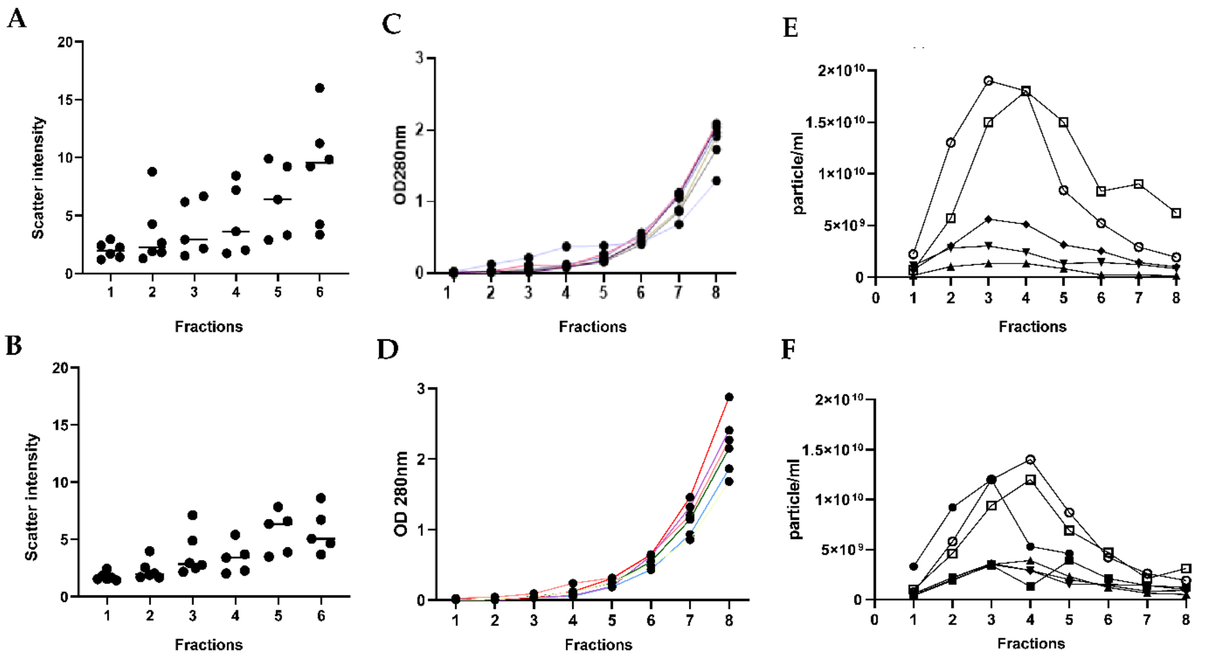

Supplementary Figure S5. Characterization of sEVs isolated using SEC 70 nm column in both case of fresh **(B,D,F)** and frozen **(A,C,E)** PFP. (**A**–**B**): The x–axis of the graph displays fractions 1–6, while the y–axis represents the Scatter Intensity, a parameter measured by the NTA (ZetaView) instrument. We found not significant (ns) difference for all 6 fractions. (**C**–**D**): The x–axis of the graph displays fractions 1–8, while the y–axis represents the absorbance measured on 280 nm with Nanodrop. We found not significant (ns) difference in the absorbance at 280nm for all 8 fractions. (**E**–**F**): The concentrations of sEV fractions (particle/mL) measured with the NTA instrument are plotted on the y–axis and the fractions 1–8 on the x–axis. We found not significant (ns) difference in the concentration of particles in all 8 fractions.
