# Supplementary material for: Effect of the 35 nm and 70 nm Size Exclusion Chromatography (SEC) Column and Plasma Storage Time on Separated Extracellular Vesicles
Source: Curr Issues Mol Biol. 2024 May 6;46(5):4337–57. doi: 10.3390/cimb46050264 (PMC11120626; doi:10.3390/cimb46050264)
Supplement: Supplementary file 1 [file cimb-46-00264-s001.zip › cimb-2955937-supplementary-Table1.pdf]

Sample Preparation

	Reagents	Storage
	NaCl-HEPES Buffer (pH: 7.0-7.4)	RT

Equipment
Desktop centrifuge, swinging bucket rotor
Microcentrifuge
pH meter

	Consumables
	3.2 mL Transfer pipette
	5.0 mL Microcentrifuge Tubes or 15 ml Conical Tubes
	1.5 mL or 2.0 mL Microcentrifuge Tubes
	0.5 mL Microcentrifuge Tubes
	Syringe Filters or 1L Bottle Top Vacuum Filter, Pore Size 0.2 µm or 0.1 µm
	Syringe Filters Pore Size 0.8 µm
	5 mL or 10 mL syringes

1. Buffer Preparation		
	1.	Use Isotonic Saline Solution 0.9% (1L) or prepare (9 g/L Sodium Chloride in distilled water)
	2.	Add a HEPES solution to a final concentration of 10 mM (use 1M HEPES solution at 100-fold dilution, add 10 mL to 1L)
	3.	Check the pH so that it is between 7.0-7.4.<br>Hydrochloric acid and/or sodium hydroxide may have been added to adjust the pH.
	4.	Filter the Buffer at 0.2 µm or 0.1 µm (use manual syringe filter or vacuum system).

2. Platelet-free plasma (PFP) Preparation		
NOTE		This description applies to 1 sampling tube (1x9mL ACD-A blood collection tube). If you plan to perform different detection techniques and/or parallel studies, it is recommended to use 2x9 mL blood samples per donor.
	1.	Check the data on the sampling tube. Record the data in your laboratory register.<br>Prepare the Sample Sheet for the day.
	2.	Check that the sample is correct. The sample is in the collection tube up to the mark (appropriate anticoagulant to blood ratio).<br>Check that the sample does not clot. Check that the sample is not hemolyzed and lipemic.
	3.	Centrifuge the blood collection tube at 2,500 g for 15 minutes.
	4.	Label a 5.0 mL, 2.0 mL and 0.5 mL microcentrifuge tube with the corresponding sample ID.
NOTE		For the centrifugation steps, you can use other volumes of tubing, but do not use polystyrene tubing. Microcentrifuge tubes with low protein binding are the most suitable for sample storage.
NOTE		For visual analysis, use paleta-scale (e.g. https://www.cdc.gov/ncezid/dvbd/pdf/Hemolysis_Palette_Bookmark-P.pdf , https://clinicalsci.info/factors-interfering-analytic-tests) . If you want to analyse quantitatively, you can use a clinical chemistry analyser, or spectrophotometric analysis to quickly assess 'indices' of haemolysis and lipemia. The general wavelength are 540 nm, 570 nm for haemolysis, and 600-700 nm for lipemia, and 450-500 nm for icterus.
Exclusion criteria		For haemolysis: intensity of the red colour of the serum and corresponding concentration of free serum haemoglobin > 1g/L.<br>For lipemia: degree of turbidity and appropriate concentration of tryglycerides > 150 mg/dL (1.7 mmol/L).<br>For icterus: intensity of yellow colour of serum and corresponding concentration of bilirubin > 1.5 mg/dL (25 µmol/L).
	5.	Use a Transfer pipette to aspirate the plasma, not the full amount, but leave about 200-300 µL. Transfer to a new tube.
NOTE		If there were 2 blood collection tubes per donor, put the plasma into one of the tubes in this step, using a 15 ml conical tube. After centrifugation, 2.8-5.5 mL of plasma is expected from 1 blood collection tube.
NOTE		If you plan to genotype, place the buffy coat in a microcentrifuge tube for DNA extraction.
	6.	Centrifuge the blood collection tube at 2,500 g for 15 minutes.
	7.	Use a Transfer pipette to aspirate the plasma, not the full amount, but leave about 50 µL. Transfer to a new tube.
	8.	Vortex the PFP samples for 5 seconds.
NOTE		This protocoll still works with a plasma volume of 2.5 mL, if you have more than one plasma then use multiple of this volume.
NOTE		If you can, you can test the platelet count in your plasma at this step. This is an optional step because it will definitely be removed in the next step. If you suspect that there are still some platelets left, or if the result shows that > $5x10^9$/L platelets are present in the sample, repeat the centrifugation step before proceeding.
NOTE		For example, if you are studying a plasma ELISA, prepare aliquot 100 µL samples in 0.5 mL tubes.
	9.	Dilute the PFP sample with NaCl-HEPES Buffer in a 1:1 ratio (2.5mL Buffer + 2.5mL PFP).
	10.	Assemble the 0.8 µm pore size syringe filter and syringe (remove the nozzle from the syringe), place it on the pre label tubes.
	11.	Vortex the diluted PFP samples for 5 seconds.
	12.	Pipette the diluted PFP sample into the syringe and wait for it to pass through the filter on its own.<br>DO NOT USE PRESSURE!
NOTE		If you see a bubble in the syringe filter, aspirate out gently with a pipette. The air bubble in the filter reduces the efficiency by reducing the available filter surface area for the sample.
NOTE		Optional: when the sample is completely filtered, but you still see a residual sample on the filter, add 500-1000 µL of Buffer to the syringe to allow the sample remaining on the filter surface to filter through.
	13.	Pipette 5.0 mL of the resulting filtered-diluted PFP sample.
	14.	Apply a snap freezing procedure (using liquid nitrogen) and store the samples at -80°C.
NOTE		If you continue the EV isolation protocol within 2 days, store the sample at 4°C.

SEC Pooled Fraction Preparation

	Reagents	Storage
	NaCl-HEPES Buffer (pH: 7.0-7.4)	RT
	0.5M NaOH	RT
	Distilled water	RT
	20% ethanol	RT
	qEVoriginal / 70 nm Legacy columns or 70 nm Gen 2 columns	Refrigerator

Equipment
Desktop centrifuge, swinging bucket rotor
Microcentrifuge
Optional: NanoDrop, Qubit, qNano, NTA

	Consumables
	5.0 mL Microcentrifuge Tubes or 15 ml Conical Tubes
	1.5 mL or 2.0 mL Microcentrifuge Tubes
	0.5 mL Microcentrifuge Tubes
	Waste container
	Syringe Filters or 1L Bottle Top Vacuum Filter, Pore Size 0.2 µm or 0.1 µm
	Syringe Filters Pore Size 0.2 µm
	5 mL or 10 mL syringes
	100kDa Ultrafiltration tube (5 ml)

**1. Buffer Preparation**		
NOTE	The preparation of the NaCl-HEPES Buffer can be read in the Samples Preparation.	
NOTE	Special care is required to prepare a solution of NaOH in water because considerable heat is liberated by the exothermic reaction.<br>Wear protective goggles and gloves as there is a chance that the NaOH soluiton may splash.<br>The concentrated NaOH solution is corrosive.	
	1.	Weigh out 10 g of NaOH into a measuring cup.
	2.	Add 250 ml distilled water. Add the powdered NaOH while stirring continuously.
	3.	Adjust to 500 ml with distilled water.
NOTE	Optional: add a pH indicator (e.g. 0.1g litmus) at the wash step for better visibility.	
	4.	Filter the Buffer at 0.2 µm or 0.1 µm (use manual syringe filter or vacuum system).
	5.	The prepared buffers should be degassed to prevent the formation of air bubbles in the resin bed.

**2. Removing the mEV**		
NOTE	If PFP samples have not been filtered to 0.8 µm, do so (see sample preparation in steps 9-13).	
	1.	Centrifuge at 18.000 g for 20 minutes at 18°C.
	2.	Label a 5.0 mL microcentrifuge tube with the corresponding sample ID.
	3.	Assemble the 0.2 µm pore size syringe filter and syringe (remove the nozzle from the syringe), place it on the pre label tubes.
	4.	With a pipette, collect the PFP sample so that it does not touch the pellet (20-30 ul may remain at the bottom of the tube).
NOTE	If the sample was still slightly lipemic, this can be seen after the centrifugation step. The plasma sample is collected on top of an opalescent layer. This "ring" is aspirated from the supernatant by pipetting so that it does not mix into the plasma sample.	
	5.	Pipette the PFP sample into the syringe and wait for it to pass through the filter on its own.<br>DO NOT USE PRESSURE!
NOTE	If you see a bubble in the syringe filter, aspirate out gently with a pipette. The air bubble in the filter reduces the efficiency by reducing the available filter surface area for the sample.	
NOTE	Optional: when the sample is completely filtered, but you still see a residual sample on the filter, add 500-1000 µL of Buffer to the syringe to allow the sample remaining on the filter surface to filter through.	
NOTE	To use the mEV samples, place the pellet at the bottom of a tube and continue cleaning.	

**3. Concentration of mEV-free plasma**		
	1.	Add 3 mL NaCl-HEPES Buffer to the Amicon 100 kDa Ultrafiltration tubes.
	2.	Centrifuge at 3.000 g for 5-10 minutes at 18 °C.
	3.	Empty the waste buffer from the bottom collector.
	4.	Add 5 mL mEV-free PFP to the Amicon 100 kDa Ultrafiltration tubes.
	5.	Centrifuge at 3.000 g for 25-40 minutes at 18 °C.
NOTE	The centrifuge spinning time depends on the type of rotor as well as the samples. Some samples may need to be centrifuged repeatedly. It is important not to over-concentrate the sample. The point of this step is to remove the buffers that help the filtration process and get back nearly the same initial sample volume. The optimal volume 1.6-1.8 mL.	
	6.	Pipette the concentrated mEV-free plasma into a 2.0 mL tube.
	7.	Pipette 100-200 µL NaCl-HEPES Buffer into the ultrafiltration tube.
	8.	Vortex gently or pipette 10 times so that the filter surface is washed, then pipette into a 2.0 mL tube.
	9.	Centrifuge the sample 10.000 g for 5-10 minutes at 16°C , acceleration/deceleration medium/low.
	10.	Pipette the sample into a new 2.0 mL tube (avoid to touch any pellets that may be produced).

**4. SEC processes**		
NOTE	This manual covers the 70 nm Legacy columns, if Gen 2 columns are used, the volumes are different!	
NOTE	Equilibrate the column before using at room temperature for 30 minutes. Check the condition of the column: no contamination and infection, no air bubbles in the resin bed, no visible cracks or the resin bed does not dry out. You can also buy the NaCl-HEPES Buffer and 0.5M NaOH.	
	1.	Required for each sample: 1x 5.0 mL or 1x15 mL, 1x 2.0 mL tube, 5x 1.5 mL tube. Mark around the sides of the tubes : 1x 5.0 mL or 1x 15 mL tube 3.0 mL volume (if you have more than one sample, then 3.0 mL, 6.0 mL, 9.0 mL, etc.) and 6x 1.5 mL tube 0.5 mL volume (or 3x 1.5mL tube 0.5mL volume and 1x 1.5mL tube 1.5mL volume). Label the 1.5 mL tubes F1-F6 and also with your sample ID.
	2.	Assemble qEV Rack
NOTE	If you have an Automatic Fraction Collector, set it according to the manufacturer's instructions.	
	Pre-Wash Step	
	3.1	Position a waste container under the column (e.g. 50 mL tube). Remove the bottom cap and allow the buffer to start flowing through the column. Allow the storage buffer to flow completely.
	3.2	Connect the buffer funnel to the top of the column.
	3.3	Rinse the column 2 times with at least 15 mL of NaCl-HEPES Buffer.
NOTE	Make sure that the column does not dry out and that the washing is continuous. When ready, replace the bottom cap.	
	Sample Collection	
	4.1	Remove the bottom cap and funnel. Continue to allow the buffer to run completely through the column.
	4.2	Remove the waste container and position the tube marked up to 3.0 mL under the column.
	4.3	Immediately pipette the prepared sample onto the loading frit.
NOTE	Carefully and accurately load the sample on the loading frit, avoid bubbles and avoid smearing the sample on the wall.	
	4.4	Reconnect the funnel to the column and fill it with 8 mL of buffer.
NOTE	If smearing has occurred, first pipette 200-500 µL of buffer onto the loading frit.	
	4.5	Once the 3.0 mL volume has been collected, continue collecting fractions into 1.5 mL tubes (0.5 mL/tube).
	4.6	Position the waste container under the column and continue to allow the buffer to flow through the column.
	Wash Step 1	
	5.1	Load the column with 15 mL of 0.5M NaOH buffer to remove protein residues.
	Wash Step 2	
	5.2	Rinse the column 2 times with at least 15 mL of NaCl-HEPES Buffer.
NOTE	If you have an additional sample, continue the process from 4.1.	
	Wash Step 3	
	6	Rinse the column 1 time with at least 15 mL of distilled water.
	Column Storage	
	7.1	Rinse the column 1 time with at least 15 mL of 20% ethanol.
	7.2	When ready, replace the bottom cap.
	7.3	Fill the column with 20% ethanol. When ready, close with the upper cap. Mark on the column how many times you have used it.
	7.4	Columns can be stored at +4 to +8 ˚C in a vertical position.
NOTE	See the manufacturer's protocol for alternative storage options.	

**5. SEC fractions quality control and pooling**		
NOTE	We recommend to check the fractions separately in a few times. Once you are convinced, you can collect the F2-F3-F4 fractions in a tube. Due to biological differences between samples, it is often the case that the F5 fraction also contains EVs. Note the relationship between EVs yield and contaminant proteins, this depends on your experimental considerations.	
NOTE	For further verification options and workflows we recommend the MISEV2023 guidelines (doi: 10.1002/jev2.12404)	
	Quantification of particle number concentration.	
	1.	Prepare dilutions of the samples, then measure it on the instrument.
NOTE	If you are using NTA techniques, the recommended dilution factors are F1: 10-50 fold; F2-F4: 100-500 fold; F5-F6: 50-250 fold. If you are using RPS techniques, the recommended dilution factors are F1: 2-5 fold; F2-F4: 5-25 fold; F5-F6: 2-10 fold.	

	Sample ID		Sample ID		Sample ID	
	protein [µg/mL]	particles/mL	protein [µg/mL]	particles/mL	protein [µg/mL]	particles/mL
F1						
**F2**						
**F3**						
**F4**						
F5						
F6						
**pool**						

	Quantification of total protein.	
	2.	Prepare dilutions of the samples, then measure it on the instrument.
NOTE	When using direct spectrophotometer techniques, do not dilute the samples. For more sensitive assays (e.g. microBCA™ Protein, Qubit™ Protein) use the manufacturer's instructions.	
Exclusion criteria	To prepare the pool, do not use a fraction with an absorbance > 1.032 (equivalent to 0.75 mg/L IgG concentration) at 280 nm and > 6.78 at 225 nm measured with a NanoDrop. Also, do not use a fraction with a particle counts < $2x10^8$ particles/mL. Scattering intensity > 10 when using an NTA instrument. For pooled samples, do not use samples with particle concentrations below 1*10^9 particles/mL.	
NOTE	Once you have determined which fractions to pooling for the pooled sample, they also recommend verifying the pooled sample.	

EV concentration

	Reagents	Storage
	NaCl-HEPES Buffer (pH: 7.0-7.4)	RT
	Molecular Biology pure water	RT

	Consumables
	Ultracentrifuge tube
	0.5 mL Microcentrifuge Tubes

Equipment
Ultracentrifuge

1. Ultracentrifugation		
	1.	Load the samples into the ultracentrifuge tube and supplement with NaCl-HEPES Buffer.
NOTE	Before loading, it is advisable to mark the side of the tube where the EV pellet is expected.	
	2.	Centrifuge the samples.
NOTE	Use the following settings with the Type-100 rotor: 100.000 g, 60 min, 4°C, accel/decel 8.<br>If you use a different type of rotor, convert the parameters (e.g. use https://www.beckman.com/centrifuges/rotors/calculator#tab2).	
	3.	Pipette off about 500 µl from the top of the sample, then pour off the buffer in one motion.
	4.	Hold the tube upside down. Pipette out any buffer left on the tube wall. Make sure you have removed it completely.
	5.	Pipette in 20-30 µl Molecular Biology pure water (or buffer, depending on what you plan to do with the sample).
	6.	Pipette the sample through the marked side at least 20 times.
	7.	Place the tube in ice or a chilled tube rack and wait at least 10 minutes.
	8.	Vortex the sample 2 times for at least 30 sec, then repeat steps 7-8.
	9.	Pipette the sample into a 0.5 mL tube.
NOTE	If you want to use MS to measure your sample it is important to use a Protein Low-Binding tube.	
	10.	If you do not proceed with further sample processing, store the sample at -80°C.

NOTE	For EV concentration you can use other methods e.g. specific surface molecules affinity methods,<br>but you have to take into account the higher EV yield loss.
