# Supplementary material for: Effect of the 35 nm and 70 nm Size Exclusion Chromatography (SEC) Column and Plasma Storage Time on Separated Extracellular Vesicles
Source: Curr Issues Mol Biol. 2024 May 6;46(5):4337–57. doi: 10.3390/cimb46050264 (PMC11120626; doi:10.3390/cimb46050264)
Supplement: Supplementary file 1 [file cimb-46-00264-s001.zip › cimb-2955937-supplementary-Table5.pdf]

"vesicular" proteins

Abbreviation	Protein Name	Extracellular exosome<br>GO: 0070062	Endomembrane system<br>GO: 0012505	Plasma membrane<br>GO: 0005886	Bounding membrane of organelle<br>GO: 0098588	35 nm n=11<br>% (A) + (B)	35 nm n=11<br>data point (A) /data point (B)<br>6/5	70 nm n=17<br>% (A) + (B)	70 nm n=17<br>data point (A) /data point (B)<br>13/4	measurement<br>(A) + (B) %	measurement<br>(A) %	measurement<br>(B) %
										35 nm = 70 nm		
										35 nm < 70 nm		
										35 nm > 70 nm		
A2MG	Alpha-2-macroglobulin	x	x			100	6/5	100	13/4			
ANXA1	Annexin A1	x	x	x	x	100	6/5	100	13/4			
ANXA2	Annexin A2	x	x	x	x	100	6/5	100	13/4			
CD5L	CD5 antigen-like			x		100	6/5	100	13/4			
G3P	Glyceraldehyde-3-phosphate dehydrogenase	x	x	x		100	6/5	100	13/4			
LG3BP	Galectin-3-binding protein	x	x			100	6/5	100	13/4			
CD34	Hematopoietic progenitor cell antigen CD34			x		18	1/1	41	6/1			
EZRI	Ezrin	x	x	x		36	1/3	41	3/4			
LAMP1	Lysosome-associated membrane glycoprotein 1	x	x	x	x	18	0/2	24	1/3			
MFGM	Lactadherin	x	x	x	x	45	2/3	88	11/4			
RAC2	Ras-related C3 botulinum toxin substrate 2	x	x	x	x	64	3/4	65	7/4			
A2ML1	Alpha-2-macroglobulin-like protein 1	x				55	1/5	59	6/4			
CD36	Platelet glycoprotein 4		x	x	x	91	5/5	94	12/4			
EF1A3	Putative elongation factor 1-alpha-like 3		x	x		82	4/5	82	10/4			
ENOA	Alpha-enolase	x	x	x	x	82	4/5	100	13/4			
GNAI2	Guanine nucleotide-binding protein G(i) subunitalpha-2	x		x		91	5/5	94	12/4			
PRDX1	Peroxiredoxin-1	x				82	4/5	94	12/4			
ACTBL	Beta-actin-like protein 2	x				45	5/0	65	11/0			
LAMB4	Laminin subunit beta-4					9	1/0	24	4/0			
CD44	CD44 antigen	x	x	x	x	27	0/3	35	4/2			
CD81	CD81antigen	x		x		73	4/4	76	10/3			
CFAH	Complement factor H	x				73	6/2	100	13/4			
ANXA4	Annexin A4	x	x	x		9	0/1	12	0/2			
CDC42	Cell division control protein 42 homolog	x	x	x	x	18	0/2	24	0/4			
CLIC1	Chloride intracellular channel protein 1	x	x	x		9	0/1	18	0/3			
FLOT1	Flotillin-1	x	x	x	x	9	0/1	12	0/2			
GNAS2	Guanine nucleotide-binding protein G(s) subunit alpha isoforms short	x	x	x		9	0/1	18	0/3			
HS90A	Heat shock protein HSP 90-alpha	x	x	x		9	0/1	18	0/3			
RAC1	Ras-related C3 botulinum toxin substrate 1	x	x	x	x	9	0/1	18	0/3			
RAP2B	Ras-related protein Rap-2b	x	x	x	x	9	0/1	12	0/2			
RAP1B	Ras-related protein Rap-1b	x	x	x	x	82	5/4	82	10/4			
HSP72	Heat shock-related 70 kDa protein 2	x	x	x	x	55	6/0	59	10/0			
RAP1A	Ras-related protein Rap-1A	x	x	x	x	45	5/0	47	8/0			
TBA1B	Tubulin alpha-1B chain					36	0/4	35	2/4			
EF2	Elongation factor 2	x	x	x		45	0/5	29	1/4			
AACT	Alpha-1-antichymotrypsin	x	x			91	5/5	88	11/4			
HSPB1	Heat shock protein beta-1	x		x		64	2/5	53	5/4			
PPIA	Peptidyl-prolyl cis-trans isomerase A	x	x			45	0/5	35	2/4			
SDCB1	Syntenin-1	x	x	x		82	4/5	76	9/4			
ACTG	Actin, cytoplasmic 2	x		x		45	0/5	24	0/4			
MYH9	Myosin-9	x	x	x		45	0/5	24	0/4			
PGK1	Phosphoglycerate kinase 1	x				45	0/5	24	0/4			
RAB10	Ras-related protein Rab-10	x	x	x	x	45	0/5	24	0/4			
RAB7A	Ras-related protein Rab-7a	x	x	x	x	45	0/5	24	0/4			
ANXA5	Annexin A5	x		x		36	0/4	24	0/4			
ANXA6	Annexin A6	x	x		x	27	0/3	18	0/3			
ARF1	ADP-ribosylation factor 1	x	x	x	x	9	0/1	6	0/1			
AT1A1	Sodium/potassium-transporting ATPase subunit alpha-1	x	x	x		36	0/4	24	0/4			
CD37	Leukocyte antigen CD37	x		x		27	0/3	24	0/4			
FLNA	Filamin-A	x	x	x		36	0/4	24	0/4			
FLOT2	Flotillin-2	x	x	x	x	27	0/3	18	0/3			
ITA6	Integrin alpha-6			x		27	0/3	24	0/4			
LDHA	L-lactate dehydrogenase A chain	x				27	0/3	18	0/3			
RAB14	Ras-related protein Rab-14	x	x	x	x	9	0/1	6	0/1			
RAB1B	Ras-related protein Rab-1B	x	x		x	9	0/1	6	0/1			
RAB8A	Ras-related protein Rab-8A	x	x	x	x	18	0/2	18	0/3			
RAB8B	Ras-related protein Rab-8B	x	x	x	x	18	0/2	12	0/2			
PRDX6	Peroxiredoxin-6	x	x			18	0/2	18	0/3			
CD9	CD9 antigen	x	x	x	x	82	5/4	59	6/4			
FLNB	Filamin-B	x		x		36	1/3	24	1/3			
PDC6I	Programmed cell death 6-interacting protein	x	x	x		55	2/4	41	3/4			
TBB2A	Tubulin beta-2A chain	x				36	3/1	35	4/2			
CD59	CD59 glycoprotein	x	x	x	x	100	6/5	76	9/4			
HSP7C	Heat shock cognate 71 kDa protein	x	x	x	x	100	6/5	88	11/4			
PRDX2	Peroxiredoxin-2	x				100	6/5	94	12/4			
STOM	Stomatin	x	x	x	x	100	6/5	94	12/4			
TFR1	Transferrin receptorprotein 1	x	x	x	x	100	6/5	94	12/4			
CDC5L	Cell division cycle 5-like protein					18	2/0	12	2/0			
TPC12	Trafficking protein particle complex subunit 12		x			9	1/0	6	1/0			
ANX11	Annexin A11	x	x			18	0/2	6	0/1			
CLH1	Clathrin heavy chain 1	x	x	x	x	27	0/3	6	0/1			
PDCD6	Programmed cell death protein 6	x	x	x	x	18	0/2	6	0/1			
RAB5C	Ras-related protein Rab-5C	x	x	x	x	18	0/2	6	0/1			
HS71A	Heat shock 70 kDa protein 1A	x	x	x		91	6/4	76	10/3			
