# Supplementary material for: Effect of the 35 nm and 70 nm Size Exclusion Chromatography (SEC) Column and Plasma Storage Time on Separated Extracellular Vesicles
Source: Curr Issues Mol Biol. 2024 May 6;46(5):4337–57. doi: 10.3390/cimb46050264 (PMC11120626; doi:10.3390/cimb46050264)
Supplement: Supplementary file 1 [file cimb-46-00264-s001.zip › cimb-2955937-supplementary-Table6.pdf]

<table>
<tr><th>category</th><th>description</th><th>term name</th><th># genes</th><th>genes</th><th># background genes</th><th>FDR value</th><th>p-value</th><th>transferred FDR value</th><th>network.SUID</th></tr>
<tr><td>GO Cellular Component</td><td>Extracellular exosome</td><td>GO:0070062</td><td>186</td><td>PSMA4|SLC2A3|*APOH* |TGM1|SERPIND1|PSMA3|PROCR|CORO1A|RETN|*PON1* |PSMA2|*C5* |PFN1|VTN |*CTSC* |*APOC3* |ARHGDIB|TPI1|LTF|HRG|*APOB* |*APOA1* |CAT|*VASP* |GGT1|*SNAP23* |KRT6B|*APOE* |BPIFB1 |CAPN7|LGALS3|IL1RN|GGH|*PZP* |*VWF* |PSMA6|BLMH|*CDH1* |ATP5F1B|PSMB1|SLC4A1|A1BG|PACSIN2 |*C6* |*CP* |ICAM1|*HPX* |IDE|ITIH4|*SERPINB13* |S100A11|ITIH1|AHSG|*SERPING1* |RHOC|ATP1B3|*PSMB4* |*PIP* |CSTB|AZGP1|*NCSTN* |COL6A3|S100P|CAMP|FABP5|TPP1|COL6A2|GUSB|GNB2|*FGB* |*PLG* |AQP1|CST6 |TOLLIP|CALML3|TLN1|STX4|PKM|SERPINF2|*TUFM* |PLEC|HSPA5|MDH2|DEFA3|*SERPINA5* |*BSG* |*FGG* |PPL |NCCRP1|*APOD* |CTSB|ALDH9A1|*FN1* |*APOA4* |SYNE2|ITIH2|VCP|HLA-DRB1|*C8A* |PNP|*TGM2* |*AGT* |H3-3A| *SERPINC1* |*APOA2* |F11R|*TAGLN2* |S100A14|*S100A16* |S100A8|S100A9|*DMBT1* |PSMA7|*GNG12* |CTSA |CAP1|*YWHAB* |PSMB2|EIF6|TXN|SLC44A1|HLA-C|SLC3A2|PODXL|LGALS7|DSP|SERPINB1|*C1QTNF3* |ZG16B|FSCN1|NME2|*CNP* |JUP|PROS1|*PSAP* |HLA-DRA|YWHAZ|HLA-A|ITGA4|ITGB2|LCP1|MASP2|NUCB1 |PCYOX1|ARPC4|IDH1|TKT|ORM2|*C4A* |HLA-B|PTPRJ|GNA13|DCD|PTPRC|*LRRC15* |PSMA1|*C4B* |*SERPINA1* |SLC2A1|*SELENOP* |WDR1|LYN|*IGLL5* |CAPN1|CFL1|*FCGR3B* |MUC5B|*JCHAIN* |*HPR* |MYL6|*IGKV2D-28* |ITGB3|PECAM1|TTN|KPRP|H4C1|CIB1|SMR3B|*IGKV1D-33* |PSMB3|*HBB* |GSS|*RDX* |ITGAM|B2M|SPN</td><td>2096</td><td>9,45E-102</td><td>7,55E-08</td><td>0,56161846</td><td>218</td></tr>
</table>
