# Supplementary material for: Effect of the 35 nm and 70 nm Size Exclusion Chromatography (SEC) Column and Plasma Storage Time on Separated Extracellular Vesicles
Source: Curr Issues Mol Biol. 2024 May 6;46(5):4337–57. doi: 10.3390/cimb46050264 (PMC11120626; doi:10.3390/cimb46050264)
Supplement: Supplementary file 1 [file cimb-46-00264-s001.zip › cimb-2955937-supplementary-Table7.pdf]

Abbreviation	Protein Name	Cellular Component											Biological Process						35 nm n=9	70 nm n=9	measurement		
		Extracellular exosome	Plasma membrane	Cell surface	Bounding membrane of organelle	Cytoplasmic vesicle membrane	COPII-coated ER to Golgi transport vesicle	Endoplasmic reticulum	Endosome	Lysosome	Cytoskeleton	Proteasome complex	Regulation of immune system process	Regulation of cell death	Regulation of cell migration	Regulation of cell-cell adhesion	Regulated of vesicle-mediated transport	Regulation of cell morphogenesis	data point (A) / data point (B)	data point (A) / data point (B)	(A) + (B) %	(A) %	(B) %
		GO: 0070062	GO: 0005886	GO: 0009986	GO: 0098588	GO: 0030659	GO: 0030134	GO: 0005783	GO: 0005768	GO: 0005764	GO: 0005856	GO: 0000502	GO: 0002682	GO: 0010941	GO: 0030334	GO: 0022407	GO: 0060627	GO: 0022604	5/4	5/4			
DRB	HLA class II histocompatibility antigen, DRB1 beta chain	x	x	x	x	x	x	x	x	x	x		x			x			0/2	0/3			
DRA	HLA class II histocompatibility antigen, DR alpha chain	x	x	x	x	x	x	x	x	x			x			x			0/2	0/3			
HLAA	HLA class I histocompatibility antigen, A alpha chain	x	x	x	x	x	x	x	x				x			x			0/4	0/4			
HLAB	HLA class I histocompatibility antigen, B alpha chain	x	x	x	x	x	x	x	x				x						0/4	0/4			
HLAC	HLA class I histocompatibility antigen, C alpha chain	x	x	x	x	x	x	x	x										0/2	0/2			
B2MG	Beta-2-microglobulin	x	x	x	x	x	x	x	x				x			x	x		0/1	2/3			
STX4	Syntaxin-4	x	x	x	x	x			x				x	x	x		x		0/1	0/2			
ITB3	Integrin beta-3	x	x	x	x	x							x	x	x		x	x	3/3	4/4			
PTPRJ	Receptor-type tyrosine-protein phosphatase eta	x	x	x	x	x							x		x		x		0/2	0/3			
ITAM	Integrin alpha-M	x	x	x	x	x							x	x			x		0/2	0/1			
ITB2	Integrin beta-2	x	x	x	x	x							x	x		x	x	x	0/3	0/3			
LEG3	Galectin-3	x	x	x	x	x							x	x	x	x	x		1/1	2/1			
PTPRC	Receptor-type tyrosine-protein phosphatase C	x	x	x	x	x							x	x	x	x	x		0/3	0/3			
4F2	4F2 cell-surface antigen heavy chain	x	x	x	x					x									0/3	0/3			
BIP	Endoplasmic reticulum chaperone BiP	x	x	x				x						x	x				0/3	0/4			
CATB	Cathepsin B	x	x	x					x	x				x					1/2	0/3			
HRG	Histidine-rich glycoprotein	x	x	x					x	x			x	x	x				3/4	2/4			
TRFL	Lactotransferrin	x	x	x					x				x	x					4/3	5/3			
TLN1	Talin-1	x	x	x							x								0/3	0/4			
EPCR	Endothelial protein C receptor	x	x	x							x								3/0	3/0			
ATPB	ATP synthase subunit beta, mitochondrial	x	x	x											x				0/3	0/3			
ICAM1	Intercellular adhesion molecule 1	x	x	x									x	x	x				0/1	0/3			
IDE	Insulin-degrading enzyme	x	x	x															0/4	0/4			
ITA4	Integrin alpha-4	x	x	x									x	x	x	x			0/2	0/2			
LEUK	Leukosialin	x	x	x									x			x			3/4	5/4			
MASP2	Mannan-binding lectin serine protease 2	x	x	x									x				x		2/2	5/3			
ZA2G	Zinc-alpha-2-glycoprotein	x	x	x															5/4	5/4			
PACN2	Protein kinase C and casein kinase substrate in neurons protein 2	x	x		x	x			x		x						x		2/0	0/0			
COR1A	Coronin-1A	x	x		x	x			x		x		x	x	x	x	x	x	0/1	0/4			
FABP5	Fatty acid-binding protein 5	x	x		x	x				x									5/4	5/4			
LYN	Tyrosine-protein kinase Lyn	x	x		x	x				x			x	x	x	x	x		0/1	0/2			
DESP	Desmoplakin	x	x		x	x					x								5/4	5/4			
GTR3	Solute carrier family 2, facilitated glucose transporter member 3	x	x		x	x													0/2	0/2			
PECA1	Platelet endothelial cell adhesion molecule	x	x		x	x									x				0/3	2/3			
CATA	Catalase	x	x		x			x		x				x					5/4	5/4			
SYNE2	Nesprin-2	x	x		x			x			x				x				2/0	2/0			
PROS	Vitamin K-dependent protein S	x	x		x			x											4/4	5/4			
GBB2	Guanine nucleotide-binding protein G(I)/G(S)/G(T) subunit beta-2	x	x		x					x									0/0	0/4			
GTR1	Solute carrier family 2, facilitated glucose transporter member 1	x	x		x						x								0/4	0/4			
PLEC	Plectin	x	x		x						x								0/4	0/4			
CTL1	Choline transporter-like protein 1	x	x		x														0/2	0/1			
CIB1	Calcium and integrin-binding protein 1	x	x					x			x		x	x	x			x	0/1	0/3			
RHOC	Rho-related GTP-binding protein RhoC	x	x					x			x				x			x	0/1	0/2			
CO6A2	Collagen alpha-2(VI) chain	x	x					x											5/4	5/4			
CO6A3	Collagen alpha-3(VI) chain	x	x					x											5/4	5/4			
VTNC	Vitronectin	x	x					x							x		x		4/4	5/4			
CAP1	Adenylyl cyclase-associated protein 1	x	x							x	x								0/1	0/2			
CAN1	Calpain-1 catalytic subunit	x	x							x									0/4	0/4			
PCYOX	Prenylcysteine oxidase 1	x	x							x									0/2	0/2			
COF1	Cofilin-1	x	x								x			x				x	2/2	3/4			
PLSL	Plastin-2	x	x								x								0/3	0/3			
PLAK	Junction plakoglobin	x	x								x				x				5/4	5/4			
PEPL	Periplakin	x	x								x								0/0	0/3			
FSCN1	Fascin	x	x								x								0/2	0/1			
TITIN	Titin	x	x								x								5/0	5/0			
WDR1	WD repeat-containing protein 1	x	x								x							x	0/1	0/3			
B3AT	Band 3 anion transport protein	x	x								x								4/4	3/4			
PODXL	Podocalyxin	x	x								x				x	x			3/3	5/4			
S10A8	Protein S100-A8	x	x								x			x					5/4	5/4			
S10A9	Protein S100-A9	x	x								x			x					5/4	5/4			
CYTM	Cystatin-M	x	x																0/2	0/3			
TGM1	Protein-glutamine gamma-glutamyltransferase K	x	x																5/4	5/4			
AQP1	Aquaporin-1	x	x											x	x				0/2	0/2			
AT1B3	Sodium/potassium-transporting ATPase subunit beta-3	x	x																0/2	0/4			
GGT1	Glutathione hydrolase 1 proenzyme	x	x										x						2/0	3/0			
GNA13	Guanine nucleotide-binding protein subunit alpha-13	x	x												x			x	0/1	0/3			
IL1RA	Interleukin-1 receptor antagonist protein	x	x													x			0/2	0/0			
ITIH4	Inter-alpha-trypsin inhibitor heavy chain H4	x	x																2/3	3/4			
JAM1	Junctional adhesion molecule A	x	x													x		x	0/2	0/3			
MUC5B	Mucin-5B	x	x																0/2	0/2			
S100P	Protein S100-P	x	x																0/2	0/2			
A2AP	Alpha-2-antiplasmin	x		x												x			0/2	0/0			
TERA	Transitional endoplasmic reticulum ATPase	x						x		x		x		x					0/2	0/2			
PPGB	Lysosomal protective protein	x						x		x									0/3	0/2			
FETUA	Alpha-2-HS-glycoprotein	x						x									x		0/1	0/3			
HEP2	Heparin cofactor 2	x						x											0/2	0/1			
ITIH2	Inter-alpha-trypsin inhibitor heavy chain H2	x						x											3/4	5/4			
KPYM	Pyruvate kinase PKM	x						x											4/4	4/4			
NUCB1	Nucleobindin-1	x						x											0/2	0/1			
TKT	Transketolase	x						x											0/2	0/4			

measurement legend: 35 nm = 70 nm; 35 nm < 70 nm; 35 nm > 70 nm

Abbreviation	Protein Name	Cellular Component											Biological Process						35 nm n=9	70 nm n=9	measurement		
		Extracellular exosome GO: 0070062	Plasma membrane GO: 0005886	Cell surface GO: 0009986	Bounding membrane of organelle GO: 0098588	Cytoplasmic vesicle membrane GO: 0030659	COPII-coated ER to Golgi transport vesicle GO: 0030134	Endoplasmic reticulum GO: 0005783	Endosome GO: 0005768	Lysosome GO: 0005764	Cytoskeleton GO: 0005856	Proteasome complex GO: 0000502	Regulation of immune system process GO: 0002682	Regulation of cell death GO: 0010941	Regulation of cell migration GO: 0030334	Regulation of cell-cell adhesion GO: 0022407	Regulation of vesicle-mediated transport GO: 0060627	Regulation of cell morphogenesis GO: 0022604	data point (A) / data point (B) 5/4	data point (A) / data point (B) 5/4	(A) + (B) %	(A) %	(B) %
TPP1	Tripeptidyl-peptidase 1	x							x	x									0/2	0/1			
ILEU	Leukocyte elastase inhibitor	x							x	x									0/2	0/2			
A1AG2	Alpha-1-acid glycoprotein 2	x								x			x						4/4	3/4			
BGLR	Beta-glucuronidase	x								x									4/0	5/0			
DEF3	Neutrophil defensin 3	x								x									3/3	4/4			
GGH	Gamma-glutamyl hydrolase	x								x									0/4	0/4			
RETN	Resistin	x								x									0/2	0/3			
TOLIP	Toll-interacting protein	x								x									0/4	0/2			
PSA1	Proteasome subunit alpha type-1	x									x	x	x						0/3	0/4			
ARPC4	Actin-related protein 2/3 complex subunit 4	x									x								0/2	0/3			
MYL6	Myosin light polypeptide 6	x									x								0/0	0/2			
CAN7	Calpain-7	x									x				x				0/0	4/0			
GDIR2	Rho GDP-dissociation inhibitor 2	x									x				x				0/1	0/3			
IF6	Eukaryotic translation initiation factor 6	x									x		x						1/3	1/3			
K2C6B	Keratin, type II cytoskeletal 6B	x									x								0/4	0/4			
PROF1	Profilin-1	x									x				x				0/3	0/4			
PSA2	Proteasome subunit alpha type-2	x										x							0/4	0/3			
PSA3	Proteasome subunit alpha type-3	x										x							0/4	0/3			
PSA4	Proteasome subunit alpha type-4	x										x							0/1	0/3			
PSA6	Proteasome subunit alpha type-6	x										x							0/2	0/3			
PSA7	Proteasome subunit alpha type-7	x										x							0/3	0/3			
PSB1	Proteasome subunit beta type-1	x										x							0/2	0/3			
PSB2	Proteasome subunit beta type-2	x										x							0/2	0/0			
PSB3	Proteasome subunit beta type-3	x										x							0/2	0/1			
S10AE	Protein S100-A14	x											x		x				4/4	3/3			
LEG7	Galectin-7	x											x			x			3/4	4/4			
PNPH	Purine nucleoside phosphorylase	x											x			x			0/3	0/2			
BPIB1	BPI fold-containing family B member 1	x											x						0/1	0/2			
H4	Histone H4	x											x						5/4	5/4			
1433Z	14-3-3 protein zeta/delta	x												x					5/4	4/4			
NDKB	Nucleoside diphosphate kinase B	x												x					0/1	0/2			
THIO	Thioredoxin	x												x					5/4	5/4			
S10AB	Protein S100-A11	x													x				0/1	0/4			
A1BG	Alpha-1B-glycoprotein	x																	0/2	0/4			
AL9A1	4-trimethylaminobutyraldehyde dehydrogenase	x																	0/1	0/2			
BLMH	Bleomycin hydrolase	x																	3/4	2/4			
CALL3	Calmodulin-like protein 3	x																	0/0	0/2			
CAMP	Cathelicidin antimicrobial peptide	x																	5/0	1/0			
CYTB	Cystatin-B	x																	0/4	0/4			
DCD	Dermcidin	x																	5/4	5/4			
FBX50	F-box only protein 50	x																	4/4	4/4			
GSHB	Glutathione synthetase	x																	0/2	0/3			
H33	Histone H3.3	x																	3/3	2/3			
IDHC	Isocitrate dehydrogenase [NADP] cytoplasmic	x																	0/3	0/4			
ITIH1	Inter-alpha-trypsin inhibitor heavy chain H1	x																	3/1	5/3			
KPRP	Keratinocyte proline-rich protein	x																	5/4	5/4			
MDHM	Malate dehydrogenase, mitochondrial	x																	0/0	0/4			
SMR3B	Submaxillary gland androgen-regulated protein 3B	x																	3/0	5/0			
TPIS	Triosephosphate isomerase	x																	0/4	0/4			
ZG16B	Zymogen granule protein 16 homolog B	x																	1/0	2/0			

35 nm = 70 nm

35 nm < 70 nm

35 nm > 70 nm
