# Supplementary material for: Effect of the 35 nm and 70 nm Size Exclusion Chromatography (SEC) Column and Plasma Storage Time on Separated Extracellular Vesicles
Source: Curr Issues Mol Biol. 2024 May 6;46(5):4337–57. doi: 10.3390/cimb46050264 (PMC11120626; doi:10.3390/cimb46050264)
Supplement: Supplementary file 1 [file cimb-46-00264-s001.zip › cimb-2955937-supplementary-Tables 2-4.pdf]

Supplementary Tables

Supplementary Table1. This table shows our step by step protocol utilized for the isolation of EVs was employed in our study.

see in PDF in Supplementary Table Figures folder

Supplementary Table2. MicroBCA results for Western blot.

**Column**	**Fraction**	**Protein Conc. (μg/μL)**
35 nm	1	0.0044
	2	0.0237
	3	0.0635
	4	0.2380
	5	0.2939
	6	0.3056
	7	0.7657
	8	too high
70 nm	1	0.0035
	2	0.0053
	3	0.0075
	4	0.0095
	5	0.0973
	6	0.1322
	7	0.3044
	8	0.6897

Supplementary Table3. Antibodies for Western blot, which used in the study.

Antibody's name	Manufacturer	Cat. number	Lot number	Conc. (mg/mL)	Dilution
Anti−CD81	Sigma	HPA0072340	C117188	0.300	1:1000
Anti−ALIX	Sigma	HPA011905	000009374	0.200	1:1000
Anti−ApoA1	Abcam	AB52945	GR3256621−2	0.159	1:750
Anti−rabbit IgG−HRP	Abcam	ab205718	GR3234362−1	2.000	1:25000
Anti−mouse IgG−HRP	BioRad	1705047	64147694	0.800	1:30000
Anti−Albumin	Abcam	ab207327	GR3213416−5	0.643	1:2000
Anit−hu−IgGAM−HRP	Invitrogen	31418	XH3676792	0.800	1:10000

Supplementary Table4. Antibodies for TEM, which used in the study.

Antibody's name	Manufacturer	Cat. number
Anti–CD9	Abcam	ab92726
Goat anti–rabbit IgG 5 nm gold	Sigma	G7277

Supplementary Table5. Table containing the "vesicular" proteins identified through quantification using the ExoCarta top 100 list across all samples. The table encompasses various analyses and additional information. The analysis of "vesicular" proteins on the basis of which the STRING figure was constructed. A) series of measurements were performed on a Bruker Maxis II Q–TOF mass spectrometer coupled to a Dionex Ultimate 3000 RSLC nanoUHPLC. For the B) series of measurements, a Thermo Scientific UltiMate 3000 nanoUHPLC was coupled to a Thermo Scientific Exploris 240 mass spectrometer. MS was performed on n=11 samples isolated from the 35 nm column, which was n=17 for the 70 nm column. Data points A and B show the number of quantified proteins in the two series of measurements. The colour scale indicates the number of samples in which a given protein was quantified in each series of measurements, and the % value was used for comparison. The measurement column is marked in pink where more quantified proteins were found in the 70 nm column and in blue where more were found in the 35 nm column. Grey indicates where the proteins were found in equal numbers on both columns, black where the quantified proteins were zero in one of the measurement series.

see in PDF in Supplementary Table Figures folder

Supplementary Table6. Proteins not included in the ExoCarta top 100 list are annotated under "Extracellular exosome GO:0070062". The table includes gene abbreviations, with genes in italics indicating those we have excluded from our individual list of proteins.

see in PDF in Supplementary Table Figures folder

Supplementary Table7. The protein list is derived from potential EV proteins identified in the 9 pairs. The table displays annotations for Cellular Component and Biological Process, along with the outcomes of the analysis. A) series of measurements were performed on a Bruker Maxis II Q–TOF mass spectrometer coupled to a Dionex Ultimate 3000 RSLC nanoUHPLC. For the B) series of measurements, a Thermo Scientific UltiMate 3000 nanoUHPLC was coupled to a Thermo Scientific Exploris 240 mass spectrometer. MS was performed on n=9 samples isolated from the 35 nm column, which was n=9 for the 70 nm column. Data points A and B show the number of quantified proteins in the two series of measurements. The colour scale indicates the number of samples in which a given protein was quantified in each series of measurements, and the % value was used for comparison. The measurement column is marked in pink where more quantified proteins were found in the 70 nm column and in blue where more were found in the 35 nm column. Grey indicates where the proteins were found in equal numbers on both columns, black where the quantified proteins were zero in one of the measurement series.

see in PDF in Supplementary Table Figures folder
